# Self-healing and hyperelastic magneto-iono-elastomers through molecular confinement of magnetic anions

**DOI:** 10.1126/sciadv.adq7441

**Published:** 2025-01-01

**Authors:** Xuan Zhang, Lishu Zhang, Meilin Liu, Chin Boon Chng, Eddy Pang Yi Ler, Jinrun Zhou, Naoji Matsuhisa, Yu Jun Tan

**Affiliations:** ^1^ Department of Mechanical Engineering, College of Design and Engineering, National University of Singapore, Singapore 117575, Singapore.; ^2^ Peter Grünberg Institut and Institute for Advanced Simulation, Forschungszentrum Jülich, 52425 Jülich, Germany.; ^3^ Department of Materials Science and Engineering, College of Design and Engineering, National University of Singapore, Singapore 117575, Singapore.; ^4^ Research Center for Advanced Science and Technology, The University of Tokyo, Meguro, Tokyo 153-8505, Japan.; ^5^ Centre for Additive Manufacturing (AM.NUS), National University of Singapore, Singapore 117602, Singapore.

## Abstract

Magneto-responsiveness in living organisms, exemplified by migratory birds navigating vast distances, offers inspiration for soft robots and human-computer interfaces. However, achieving both high magneto-responsiveness and resilient mechanical properties in synthetic materials has been challenging. Here, we develop magneto-iono-elastomers (MINEs), combining exceptional magnetization [2.6 emu (electromagnetic units)/g] with hyperelasticity and self-healability. Such a MINE consists of a magnetic ionic liquid (MIL; [Emim][FeCl_4_]) and a urethane group–based polymer that can distinctively confine magnetic anions through strong intermolecular interactions, including potential hydrogen bonds and metal-coordination bonds. This confinement enables high MIL loading (80 wt %) while maintaining structure integrity, resulting in a high ionic conductivity exceeding 10^−3^ S/cm. Furthermore, the synergistic interplay of these reversible bonds in MINEs contributes to an outstanding elastic recovery that surpasses 99%, alongside good self-healing capabilities. The unique combination of these attributes positions MINE as a promising candidate for diverse magnetoelectronic applications, encompassing wearable strain sensors, contactless magneto-responsive electronics, see-through touch panels, and soft magnetic carriers.

## Teaser

A material combining high magnetization, hyperelasticity, and self-healing properties is achieved by magnetic anion confinement.

## INTRODUCTION

**Fig. 1.**
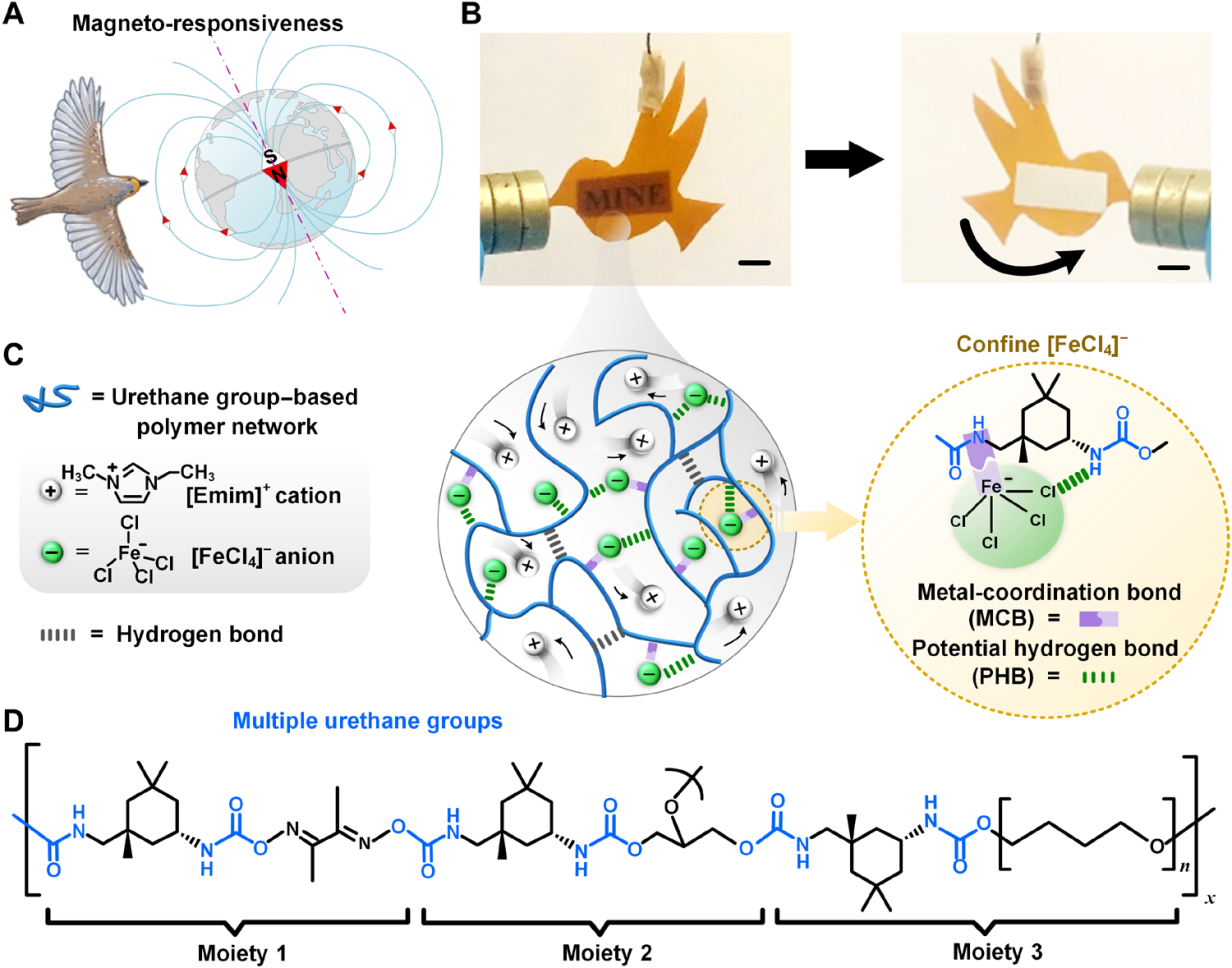
Multifunctional MINEs through molecular confinement of magnetic anions. (**A**) The magneto-responsive ability in European robins (*1*) aids them in sensing Earth’s magnetic field for migration and navigation. (**B**) Photographs of a bird-shaped transparent MINE4 with 60 wt % MILs ([Emim][FeCl_4_]), allowing for navigation oriented by an external magnet (scale bars, 1 cm). (**C**) Schematic illustration of the MINE composed of MILs incorporated into a urethane group–based polymer network. This material design is characterized by the confined magnetic anions within the polymer matrix through their MCBs and PHBs, while the free cations contribute to ionic conductivity and act as mobile charge carriers. (**D**) Chemical structure of the as-synthesized polyurethane elastomer with multiple urethane groups.

Magneto-responsiveness, the ability to sense and respond to external magnetic fields, is observed in diverse living organisms, including migratory birds like the European robin (*Erithacus rubecula*). This adaptation allows them to navigate long distances, as exemplified by their continental migrations (Fig. 1A) (*1*). This phenomenon inspires the development of soft magnetic materials and magnetic fluids for emerging applications in soft robots, human-computer interfaces, and biomedical technologies (*2*–*5*). Similarly, the inherent self-healing and resilience of skins have guided the design of materials with similar properties, essential for practical applications requiring recurrent deformation and long-term durability (*6*–*8*). Traditionally, metal-based magnetic particles like Fe_2_O_3_, Fe_3_O_4_, and Nd_2_Fe_14_B are used to impart magnetism in these materials. While offering high magnetization, incorporating these particulate fillers into soft, compliant matrices inevitably results in both optical opaqueness and a stiffening effect, compromising elasticity (*9*). Magnetic ionic liquids (MILs), noteworthy for their intrinsic transparency and incorporation of magnetic metal ions (*10*), emerge as a promising alternative for maintaining softness in magneto-responsive materials. Researchers first created a transparent magnetic ionogel in 2010 by combining [Bmim][FeCl_4_] with poly(methyl methacrylate) (PMMA) (*11*). This pioneering work paved the way for further exploration, leading to the successful incorporation of various other MILs into PMMA polymer matrices (*12*).

However, achieving high magneto-responsiveness alongside soft yet resilient mechanical properties remains a notable challenge (table S1). This inherent trade-off arises because a high loading of MILs weakens the polymer network. This limitation likely stems from the weak interaction between magnetic anions and polymer chains. We hypothesize that identifying suitable combinations of elastomer matrices and MILs with favorable interactions appears critical to overcoming this hurdle.

Herein, we develop magneto-iono-elastomers (MINEs), a class of materials combining good magnetization [2.6 electromagnetic units (emu)/g] with hyperelasticity and self-healing capabilities. The material design leverages a urethane group–based polymer that effectively confines the magnetic FeCl_4_ anions, sourced from MILs, within the matrix through their intermolecular interactions, including potential hydrogen bonds (PHBs) and metal-coordination bonds (MCBs). This confinement enables a high MIL loading, reaching up to 80 wt % in MINE, allowing for the fabrication of freestanding materials. The synergistic contributions of multiple intermolecular interactions between MIL and polymer lead to a remarkable combination of properties in MINEs: transparency, ionic conductivity (more than 10^−3^ S/cm), magneto-responsiveness (Fig. 1B and fig. S1), hyperelasticity (elastic recovery of 99%), and self-healing (fig. S2). The exceptional versatility of MINEs holds promise for applications in magneto-responsive electronics, touch and strain sensors, and soft carriers.

## RESULTS

### Material design and fabrication

To create MINEs, we developed a transparent elastomer matrix rich in multiple urethane groups. These groups readily form strong intermolecular interactions with the FeCl_4_ anions of the MIL, effectively confining them within the elastomer through PHBs and MCBs (Fig. 1C). This distinct arrangement allows Emim cations to retain their free mobility under an applied alternating current, contributing to the material’s ionic conductivity. Simultaneously, the confined FeCl_4_ anions solely contribute to the material’s magnetic attributes, aligning their magnetic moments in response to an external magnetic field.

The urethane group–based polymer was synthesized through a facile one-pot polycondensation process involving four monomers [dimethylglyoxime (DMG), poly(tetramethylene ether) glycol (PTMEG), glycerol, and isophorone diisocyanate (IPDI)] (Fig. 1D and fig. S3). Increasing the content of IPDI and glycerol monomers promotes the polymer’s polymerization rate (note S1), leading to longer polymer chains with higher molecular weights. Glycerol also functions as a cross-linker to tune the polymer’s mechanical properties (fig. S4). Three types of urethane group–based polymers with varying degrees of cross-linking (low, medium, and high) were investigated. These different cross-linked polymers were then mixed with various contents of [Emim][FeCl_4_] to fabricate a range of MINEs, primarily MINE1 to MINE6 (refer to table S2 for preparation details) to explore their performance advantages. When introduced to a low–cross-linked prepolymer solution dissolved in acetone, MILs increase their molecular weight and lead to the narrow polydispersity of the resulting MINEs (fig. S5). This suggests that MIL aids in extending the polymer chain length as a Lewis acid (*13*).

MINE1, a low–cross-linked polymer containing 20 wt % [Emim][FeCl_4_], is a unique material. Unlike composites filled with nanoparticles, MINE1 is entirely particle-free, resulting in a yellowish, transparent appearance because of the excellent miscibility between the transparent urethane group–based polymer and the brown MIL (fig. S6). Notably, MINE1 exhibits high light transmittance, reaching approximately 80% at wavelengths exceeding 500 nm. In stark contrast, composites containing Fe_3_O_4_ nanoparticles suffer from both complete opacity and uneven nanoparticle distribution (fig. S7). Furthermore, MINE1 demonstrates remarkable stretchability, achieving a maximum elongation of about 1242%. This represents a substantial improvement compared to the low–cross-linked polymer, which only reaches a maximum elongation of around 450% (fig. S8, A and B).

### High MIL loading in MINEs

Achieving high MIL content within MINEs is critical for practical applications, as it ensures sufficient magneto-responsiveness and ionic conductivity. However, incorporating more than 40 wt % MIL into a low–cross-linked polymer matrix creates a sticky gel that deforms over time (fig. S9). To address this limitation, we used a urethane group–based polymer with a high cross-linking degree. This modification enables the successful incorporation of up to 80 wt % MIL while maintaining the material’s mechanical integrity. In contrast, previously reported materials can only hold up to 50 wt % MIL (*11*) before losing their self-supporting structure. This limited MIL loading has deterred further exploration of their magnetoelectronic applications. Even with the increased MIL content, the material retains its optical transparency (fig. S10).

**Fig. 2.**
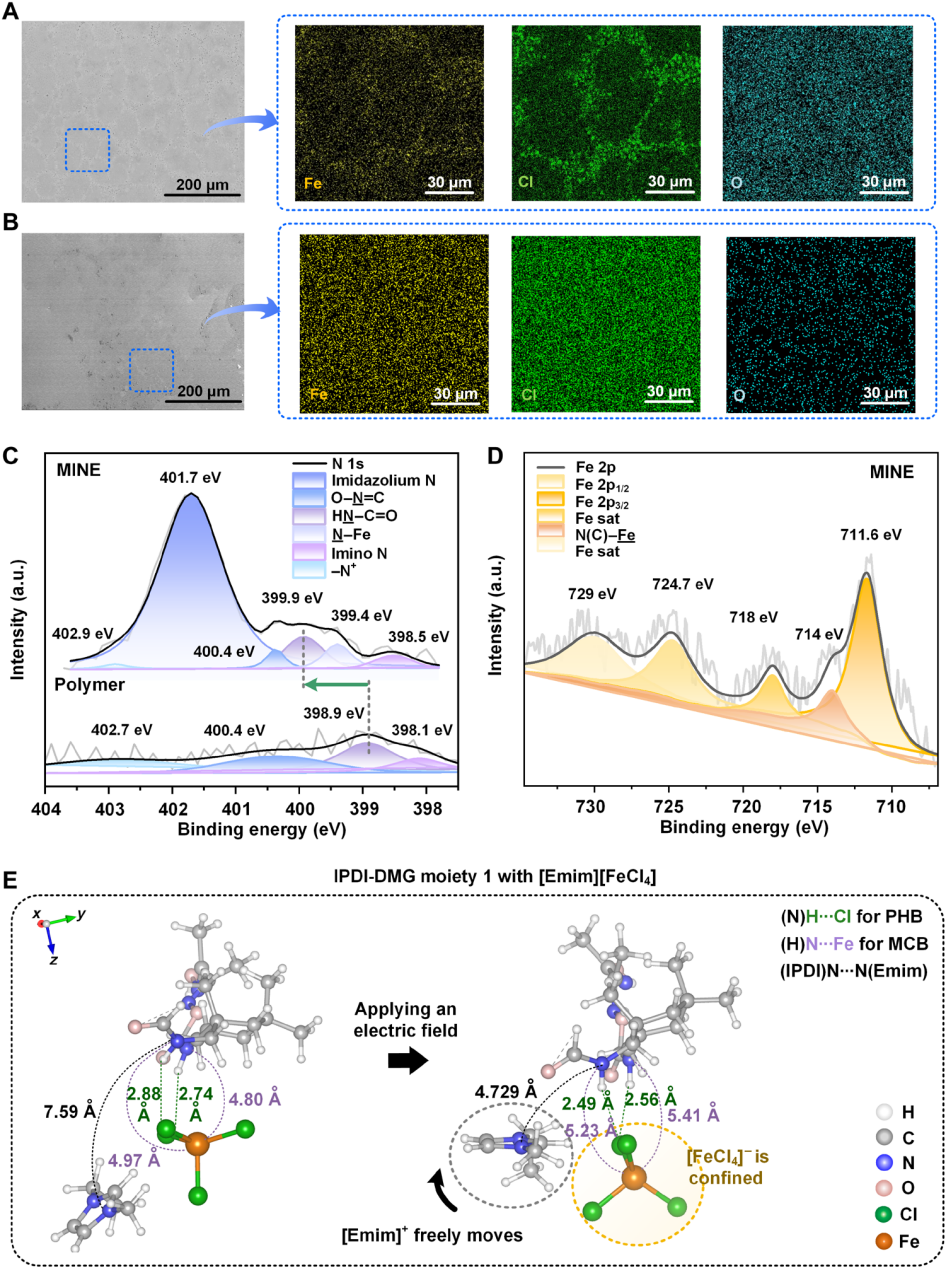
Materials characterizations and interaction mechanisms in MINEs. Scanning electron microscopy images of (**A**) MINE1 and (**B**) MINE4 and element analysis of their zoomed-in images. Results for MINE1 reveal a MIL-rich domain at the cell boundaries with a polymer-rich domain inside the cells. As the MIL concentration increased to 60 wt %, the characteristic morphology disappeared, with MIL distributed throughout the entire MINE matrix, resulting in a homogeneous mixture. High-resolution XPS spectra of (**C**) N 1s for both the polymer and MINE4, as well as (**D**) Fe 2p for MINE4. A substantial downward shift of 1 eV in the amide N (–NH–C=O) peak indicates the formation of PHBs and MCBs because of MIL incorporation. A 714-eV peak in Fe 2p (MINE4) further confirms Fe–N coordination bonds, which are absent in pure MIL. a.u., arbitrary units. (**E**) DFT calculations reveal PHBs (N–H···Cl) between IPDI-DMG moiety 1 and the [Emim][FeCl_4_] complex, as shown by interatomic distances falling within accepted hydrogen bonding ranges. Reoptimizing structures under a 0.1-V/Å electric field demonstrates a stronger impact on the Emim cation’s movement than that of the FeCl_4_ anion, suggesting the anions’ effective confinement because of both PHBs and MCBs within the MINE system.

Further insights into the material’s characteristics and morphology are gained through x-ray diffraction (XRD), thermogravimetric analysis (TGA), and scanning electron microscopy observations, showing that MINEs are amorphous (fig. S11) and homogeneous with a decomposition temperature of around 159°C (fig. S12). In MINE1, cell-like structures were visible, revealing a MIL-rich domain at the cell boundaries with a polymer-rich domain inside the cells (Fig. 2A). As the MIL concentration increased to 60 wt %, the characteristic morphology disappeared since MIL covered the entire MINE matrix (Fig. 2B).

### [FeCl_4_]^−^ confinement through intermolecular interactions

The presence of partially charged regions on the urethane group–based polymer, revealed by its electrostatic potential map (note S2), suggests weak ion-dipole interactions with MIL. The interactions assist the polymer in encapsulating all ionic species within its matrix. However, when fabricating a control material by blending the polymer with a common ionic liquid ([Emim][TFSI]), the resulting material is opaque due to visible phase separation (fig. S13). Density functional theory (DFT) calculations have also revealed stronger interactions between the polymer and [FeCl_4_]^−^ than with [TFSI]^−^ (note S3).

**Fig. 3.**
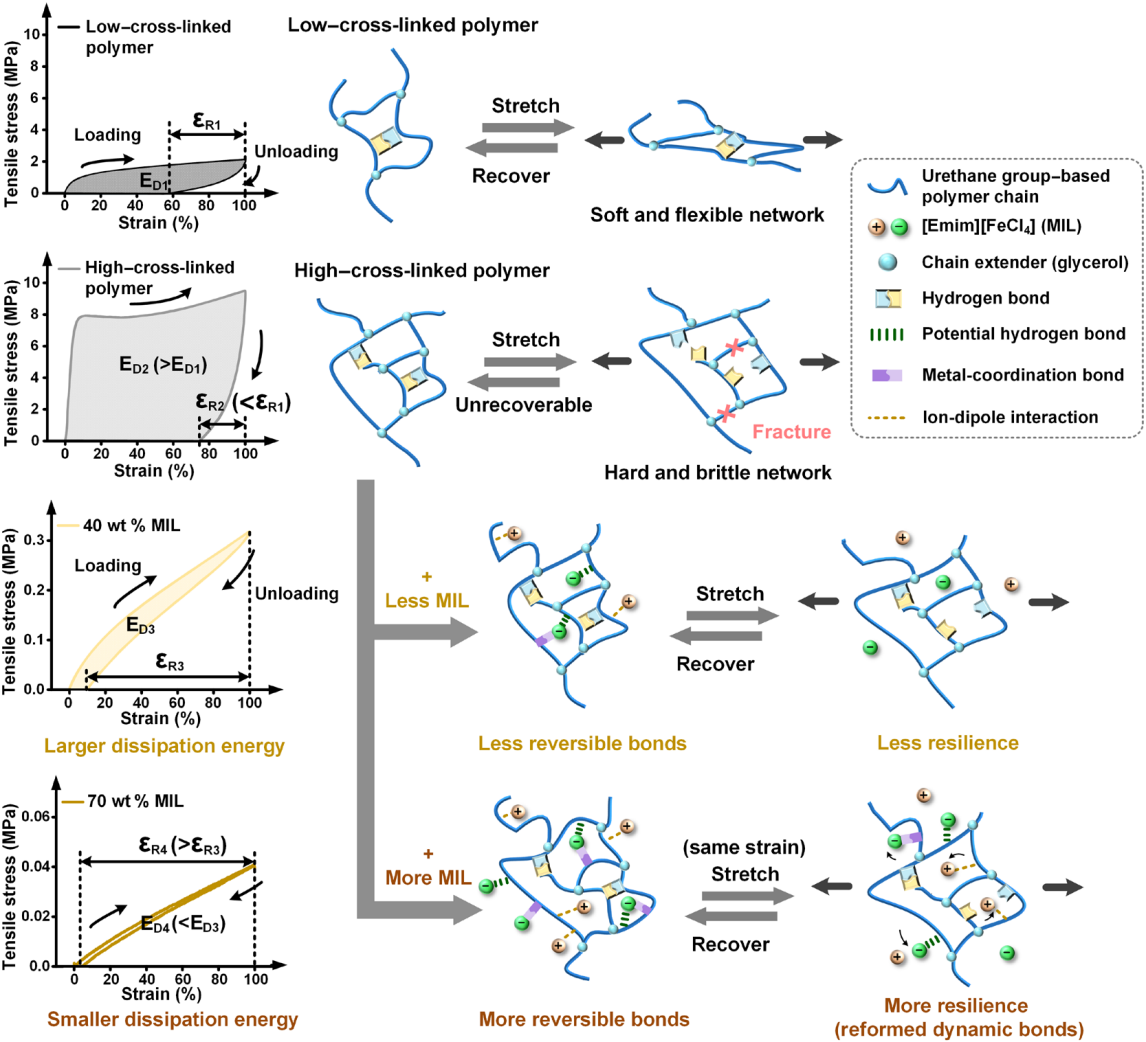
Schematic comparison of the resilient properties between different urethane group–based polymers and self-healable MINEs. This diagram explains the achievement of both hyperelasticity and self-healing in MINEs because of the presence of multiple reversible bonds (PHBs, MCBs, and ion-dipole interactions). These intermolecular dynamic bonds readily break and reform under applied and released stress. The facile reformation of these broken bonds at damaged material interfaces underpins the intrinsic self-healing capacity of MINEs. Notably, as the polymer cross-linking degree increases, the formation of additional urethane groups facilitates higher MIL loading and, consequently, a greater number of reversible bonds. MINEs with higher MIL content exhibit lower dissipation energy ($E_{D4}$) at similar strain levels and greater elastic recovery ($\varepsilon_{R4}$) after deformation, indicating superior resilience. Note that $\varepsilon_{R}$ and $E_{D}$ represent the normalized elastic recovery and dissipation energy, respectively. Their detailed definitions are provided in note S5.

Through further investigation, Fourier transform infrared spectroscopy–attenuated total reflectance (FTIR-ATR) tests suggest the potential for the formation of PHBs between amide units of the urethane group–based polymer and Cl atoms of MIL anions (fig. S14). Unlike conventional hydrogen bonds, PHBs involve electronegative halides of metal complex anions and the neighboring cations. Such PHBs have been identified within paramagnetic ionic liquids (*14*). Furthermore, x-ray photoelectron spectroscopy (XPS) spectra reveal the presence of PHBs and MCBs involving the FeCl_4_ anion and the polymer backbone (Fig. 2C and fig. S15). The incorporation of MIL induces a substantial peak downward shift of 1 eV in the amide N (–NH–C=O) peak observed in the spectrum (*15*). The appearance of additional peaks at 399.4 eV in the N 1s spectrum and at 714 eV in the Fe 2p spectrum of MINE strongly suggests the formation of Fe–N coordination bonds (MCBs) (*16*). DFT calculations in note S4 further validate multiple PHBs between C–H or N–H in the polymer moieties and Cl in [FeCl_4_]^−^. Most interatomic distances fall within the accepted range for hydrogen bonding (2.7 to 3.3 Å) (*17*) or even closer. While the strength of each PHB within the MINE might be lower compared to traditional hydrogen bonds, the cumulative presence of multiple PHBs contributes to a strong intermolecular force. Consequently, the high–cross-linked polymer, rich in more urethane groups, facilitates the formation of PHBs and MCBs between the polymer and MIL components more effectively. The enhanced interactions allow the material to achieve a high MIL loading of up to 80 wt %. Moreover, both hyperelasticity and self-healing properties of MINEs also stem from these strong, dynamic, and revisable intermolecular bonds between the MIL and the polymer, complemented by the synergistic effects of hydrogen bonding and oxime-urethane bonds within the polymers themselves (Fig. 3).

A common electric field with a magnitude of 0.1 V/Å has been applied in DFT calculations to investigate ion mobility under electrical stimulation (Fig. 2D and note S6). The electric field induces notably greater displacement of the bulkier Emim cation versus the FeCl_4_ anion. This suggests that the PHBs and MCBs effectively confine FeCl_4_ anions within the urethane group–based polymer matrix, restricting their movement under the electric field. In contrast, the Emim cations exhibited higher comparative mobility in MINE attributable to weaker intermolecular ion-dipole interactions.

### Electrical and magnetic performance

**Fig. 4.**
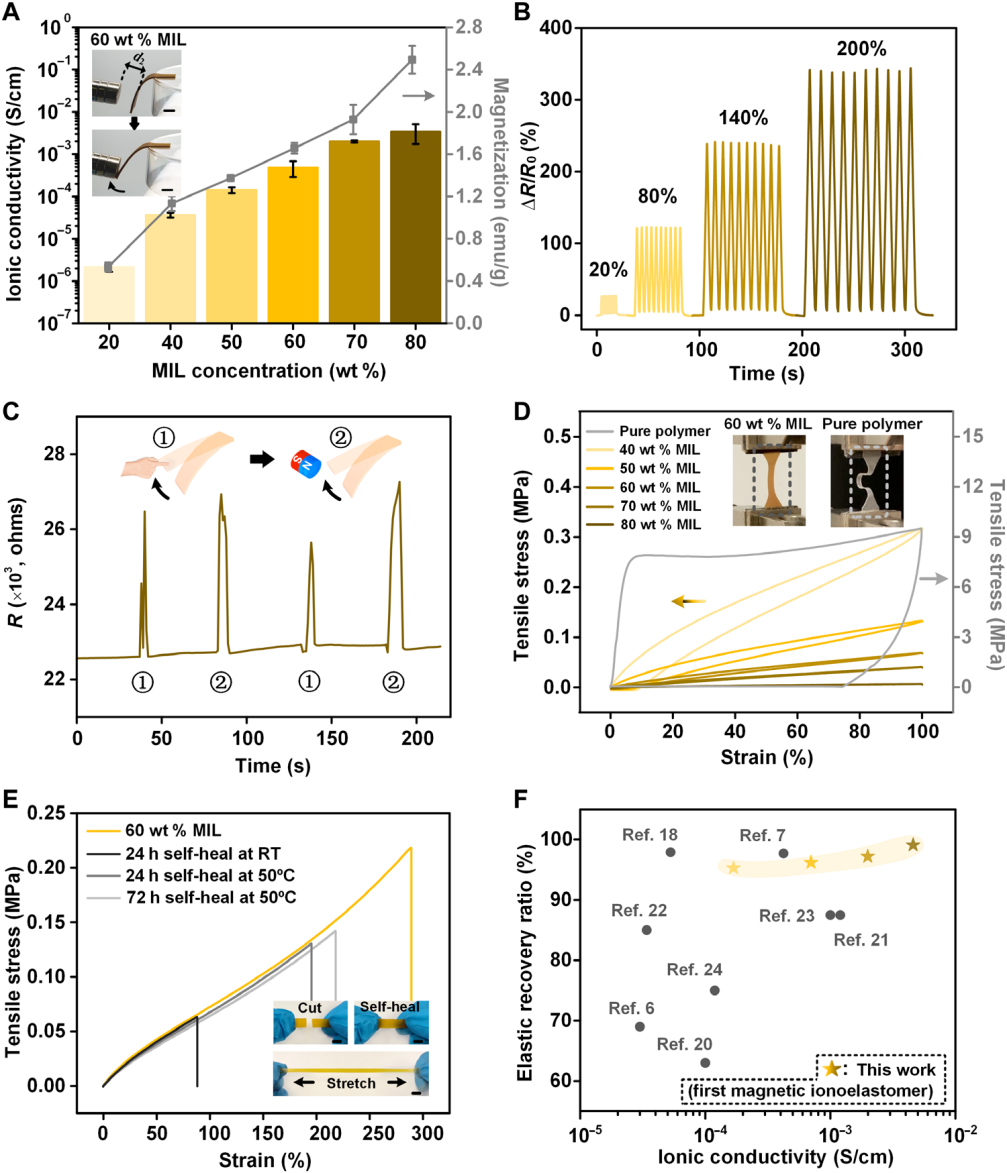
Electromagnetic, hyperplastic, and self-healable performances of MINEs. (**A**) Ionic conductivity and magnetization of MINEs with MIL contents varying from 20 to 80 wt %. Error bars indicate standard deviations from three measurements for each point. Inset: photographs of magneto-responsive actuation of MINEs containing 60 wt % MIL (scale bars, 5 mm). (**B**) Resistance changes of MINE4 under stretch-release cycles at strain percentages from 20 to 200%, with an overhead speed of 15 mm/s. (**C**) Resistance changes of MINE4 bent by hand or an external magnet, showcasing the potential as contactless, magnetically actuated strain sensors. (**D**) First-cycle loading-unloading curves of urethane group–based polymer and various MINEs. With increasing MIL content, the size of the hysteresis loop decreases, indicating greater elasticity. Inset: photographs of the polymer and MINE4 after 10 stretching-releasing cycles. (**E**) Self-healing performance of MINE4 under various healing conditions. h, hours. Inset: self-healing performance of MINE at RT (scale bars, 5 mm). The reconnected and self-healed cut damage on the MINE remained stretchable. (**F**) Comparison between the elastic recovery ratio and the ionic conductivity of the MINEs and other self-healing, transparent ionic elastomers. All MINEs exhibit magneto-responsiveness, a trait not found in the other ionic elastomers.

Both the ionic conductivity and magnetization of MINEs exhibit a concomitant increase with higher MIL loading (Fig. 4A and fig. S16). At 80 wt % [Emim][FeCl_4_] content, the MINE achieves an ionic conductivity exceeding 10^−3^ S/cm, representing an approximately 1300-fold enhancement compared to MINE1 (containing 20 wt % MIL). MINE6, containing 80 wt % MIL, also exhibits a magnetization of around 2.6 emu/g (fig. S17). Notably, MINE4 (high–cross-linked polymer containing 60 wt % MIL) shows improved magneto-responsive actuation compared to its 20 wt % MIL counterparts, highlighting its potential for noncontact shape morphing applications (movie S1).

Figure S18 demonstrates the multifunctional advantages of MINE, combining optical transparency, ionic conductivity, and magneto-responsiveness. Under external magnetic actuation, MINE4 could not only illuminate a light-emitting diode (LED) but also allow light transmission after completing a circuit. In contrast, the control material based on Fe_3_O_4_ nanoparticles was inherently nonconductive and light blocking (movie S2).

Moreover, the electromagnetic performance of MINEs under dynamic processes has been investigated. Figure 4B depicts the change in resistance of MINE4 during repeated stretch-release cycles at different strain ratios. These reliable outputs demonstrate the applicability of MINE4 as a strain sensor with exceptional sensitivity. Its gauge factor is 1.45 at low strains (0 to 60%) and 1.89 at high strains (60 to 200%) (fig. S19). Even under exposure to external magnetic fields, the electrical response remained extremely stable, showing negligible deviation from its intrinsic output (fig. S20). This resilience to external disturbances also extends to its magneto-responsiveness, as evidenced by the sensor’s consistent performance under various electric fields applied (figs. S21 and S22).

Crucially, MINEs have the unique ability to decouple their magnetic and electrical properties, preventing them from interfering with each other (Fig. 4C). This characteristic makes them highly promising for applications requiring independent control of both properties, such as magnetically actuated strain sensors. Unlike traditional magnetic particle–filled materials, which often suffer from an indistinguishable signal-to-noise ratio when placed near a magnet, MINE4 exhibits a consistent change in resistance without generating additional noise signals during magnetic actuation (fig. S23). This translates directly into a clean strain signal, rendering MINEs ideal for contactless strain-sensing applications. The success of this approach hinges on the intricate material design, which effectively confines magnetic anions within the elastomer matrix.

### Resilience and self-healing

Typically, incorporating substantial amounts of ionic liquids into polymer matrices weakens both the modulus and resilience of the resulting material because of their plasticizing effect (*6*, *18*). This plasticization enhances the mobility of polymer chains, hindering the material’s ability to regain its original shape. As expected, increasing the amount of MIL in MINEs leads to a reduction in both tensile strength and modulus (fig. S24A). However, we observed a notable improvement in the resilience of MINEs with increasing MIL content (Fig. 4D and fig. S25). Refer to note S5 for a detailed description of resilience. The smaller hysteresis loop observed in the cyclic tensile curve indicates lower energy dissipation, reflecting high elasticity. MINE6 exhibits an impressive 99% elastic recovery ratio after 100% strain. Notably, the high–cross-linked polymer lacked rapid strain recovery after stretching. Conversely, the incorporation of MIL enabled MINE4 to recover its original dimensions promptly even after being stretched to 100% strain (movie S3). All MINEs with different MIL contents were characterized using the Ogden hyperelastic model (figs. S24, B to D).

Movie S4 further highlights this phenomenon, illustrating how a higher MIL content in MINEs significantly enhances their resilience. The MINE containing 60 wt % MIL exhibited superior rubber-like properties compared to those with 20 wt % MIL. Upon release from ~600% strain, MINE4 swiftly reverted to its original form in less than 1 s, a recovery rate almost 20 times faster than that of MINE1. In addition, the endurance of MINEs is evidenced by their capability to withstand a thousand stretch-release cycles (fig. S26).

A urethane group–based polymer with a low degree of cross-linking exhibits autonomous self-healing at room temperature (RT). This is attributed to both intermolecular hydrogen bonding and intramolecular oxime-urethane bonds (*19*). Adding just 20 wt % MIL to this low–cross-linked polymer significantly increases its self-healing efficiency by 412% (fig. S8, A and B). After self-healing autonomously for 72 hours at RT, MINE1 could recover 88.7% of its original mechanical performance. The addition of MIL allows for better diffusion of polymer chains, leading to increased opportunities for the reformation of intermolecular and intramolecular bonds. Notably, after 360 hours, the damaged interface caused by cutting MINE1 into two pieces was almost completely healed (fig. S8C). MINEs involving higher–cross-linked polymers require weeks to recover their mechanical performances autonomously at RT when bifurcated (fig. S27A). Mild heat can speed up the self-healing process of these materials. The self-healing efficiency of MINE4 can reach 76% after heating at 50°C for 72 hours (Fig. 4E). In general, a higher MIL content leads to better self-healing performance (fig. S27B). Among the self-healable ionic elastomers reported (*6*, *7*, *18*, *20*–*24*), our material demonstrates exceptional elasticity and conductivity while having unparalleled magneto-responsiveness that is not found in other ionoelastomers (Fig. 4F).

### Magnetoelectronic applications

**Fig. 5.**
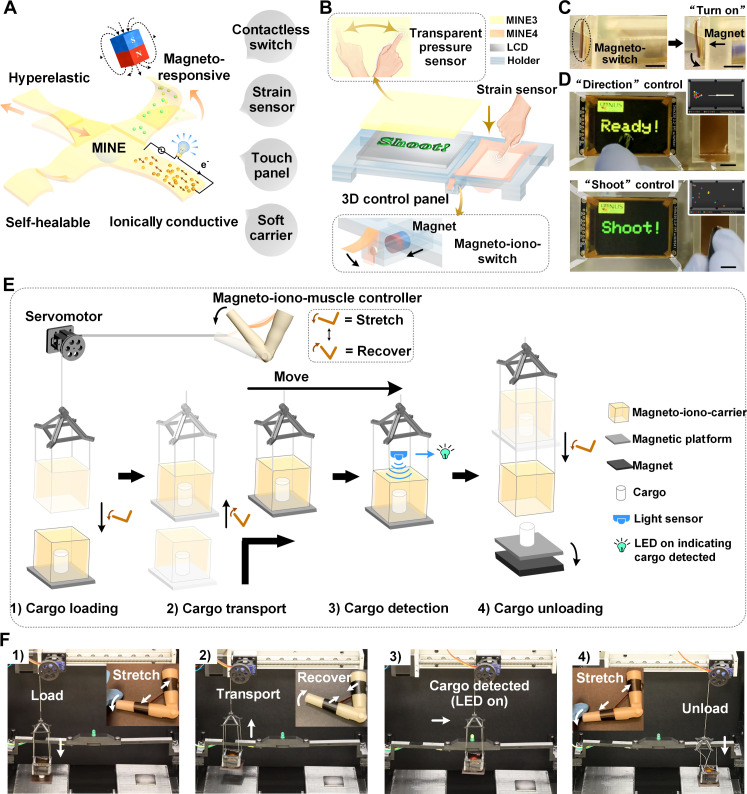
Applications of magnetoelectronics based on multifunctional MINEs. (**A**) Schematic illustration of a hyperelastic, self-healing, ionically conductive, and magneto-responsive MINE enabled by multiple intermolecular bonds between MILs and the polymer. The multifunctional MINEs enable potential applications such as contactless magnetic switches, strain sensors, soft magnetic carriers, and touch panels. (**B**) Schematic illustration of a MINE-based 3D control panel applied for the control of an 8 Ball Pool game. (**C**) Photographs of the magneto-iono-switch actuated by an external magnet with a magnetic field of around 400 mT (scale bars, 1 cm). (**D**) Screenshots of the pool game when prompted to control the direction and shooting of the ball (scale bars, 1 cm). (**E**) Schematic illustration of a soft magneto-iono-carrier system controlled by a hyperelastic magneto-iono-muscle for cargo delivery. The carrier features a robust and magneto-responsive wall for contactless magnetic latching while also showing a transparent top for light-based detection. (**F**) Screenshots of various stages of the entire setup during the delivery of a Styrofoam cargo to its destination: (1) cargo loading based on magnetic attraction and physical adhesion, (2) cargo transport, (3) cargo detection for successful pickup confirmation, and (4) cargo unloading.

MINEs demonstrate immense potential in magnetoelectronic applications because of their unique combination of high conductivity, magneto-responsiveness, hyperelasticity, and self-healing capability (Fig. 5A). Their multifunctional nature, coupled with superior surface conformality compared to traditional magnetic particle–filled materials, makes MINEs ideal strain sensors for integration into artificial smart muscles and wearable smart gloves (figs. S28 to S31). The material’s excellent self-healing ability further enhances the long-term durability and reliability of the strain sensor. Figure S32 indicates that MINE maintains consistent strain-sensing performance and magnetic properties after being severed and subsequently healed. We have successfully applied MINE to create a contactless magneto-iono-switch system to manipulate a seven-segment display (fig. S33). Each leg responds to an external magnet, activating a circuit similar to a magnetic reed switch. Microcontroller-generated signals then illuminate specific numbers on the display (table S3 and movie S5).

To comprehensively demonstrate the versatility of MINEs, we engineered a three-dimensional (3D) control panel for human-machine interaction (Fig. 5B and fig. S34). This assembly, constructed entirely from MINEs, integrates a two-dimensional (2D) transparent touch panel (MINE3), a 1D strain-sensing panel, and a magneto-iono-switch (both from MINE4) (fig. S35). The integrated setup enables control of an 8 Ball Pool game at a low voltage of 1 V (fig. S36). The 2D touch panel controls the “direction” control of the pool cue, the 1D strain sensor governs the “shoot” function, and the “on/off” function of the liquid-crystal display (LCD) is managed through a magneto-iono-switch using an external magnet with a magnetic field of 400 mT (Fig. 5, C and D, and movie S6). Leveraging the unique strengths of individual MINEs, we established a soft magneto-iono-carrier system controlled by a hyperelastic magneto-iono-muscle for efficient cargo delivery (Fig. 5, E and F, and fig. S37). The hyperelastic muscle controller regulates the vertical motions of the carrier through stretching and recovery. Combining magnetic attraction and physical adhesion, the MINE carrier and a magnetic platform can form a secure enclosed space for cargo during delivery. This “detachable tray” platform can be strategically pulled away by a strong magnet at the destination, releasing the cargo in a controlled manner. The transparent top layer of the carrier allows an overhead light sensor to detect cargo presence, triggering a green LED for successful pickup confirmation. The LED remains off in the absence of cargo, offering immediate feedback on the mission’s outcome (movie S7). Both potential applications amalgamate all the multifunctional superiorities of MINEs into one system comprehensively.

## DISCUSSION

In conclusion, this study presents a multifunctional MINE combining high magnetization (2.6 emu/g) with remarkable hyperelasticity (achieving 99% elastic recovery), alongside good self-healability. MINE is composed of a MIL ([Emim][FeCl_4_]) and a urethane group–based polymer that can effectively trap the FeCl_4_ anions through strong and reversible intermolecular interactions, including PHBs and MCBs. This trapping mechanism allows for a high amount of MIL (up to 80 wt %) to be incorporated without compromising the structure integrity of the material. Consequently, MINEs achieve superior ionic conductivity exceeding 10^−3^ S/cm while still being optically transparent. The multifunctional properties of MINEs can be tailored to specific application needs. This tunability empowers them to find utility in strain sensors, contactless magnet-actuated electronics, touch panels, and soft carriers. In contrast to the functional gels documented, our MINE demonstrates a clear superiority in their comprehensive multifunctionality and versatility (fig. S38).

The versatile MINE synthesis method can be applied to most paramagnetic ionic liquids, especially for ionic liquids containing a tetrachloroferrate anion, like [Bmim][FeCl_4_]. The MINEs with [Bmim][FeCl_4_] showed similar mechanical, electrical, and magnetic properties to those made with [Emim][FeCl_4_] (fig. S39).

Moving forward, the use of MINEs holds the promise of ushering in a multitude of pioneering applications across diverse domains. This potential stems from their high dielectric permittivity (fig. S40) and the unique characteristics inherent to MILs, including their long-range ordering at low temperatures and physiochemical properties like thermomorphism (*25*, *26*). While these MIL properties offer benefits in their native liquid state, MINEs create a previously unidentified opportunity to use these advantages in solid, elastomeric form. In particular, MINE6 exhibits magnetic properties akin to MILs, such as exhibiting a negative Curie-Weiss temperature and having a Neel temperature (*27*, *28*) (fig. S41), that are absent in MINE4. By adding a substantial amount of MIL into an elastomer, MINEs have the potential to facilitate various innovative applications, including but not limited to stretchable versions of paramagnetic ionic gating (*29*) and electrochemical sensors (*26*). As exploration into the fundamental aspects of MILs continues, elastomers containing MILs, such as MINEs, are poised to redefine the landscape of advanced materials with their unrivaled promise.

## MATERIALS AND METHODS

### Materials

Polytetrahydrofuran (polyTHF, also called poly(tetramethylene ether) glycol, *M*_n_ = ~1000 g/mol), DMG (≥99%), IPDI (98%), glycerol (≥99%), acetone (≥99.5%), and iron(II, III) oxide nanopowder (50- to 100-nm particle size, 97%) were all purchased from Sigma-Aldrich. [Emim][FeCl_4_] was purchased from IoLiTec Ionic Liquids Technologies GmbH (Germany), [BMIM][FeCl_4_] was purchased from Tokyo Chemical Industry Co. Ltd. (Japan), and [Emim][TFSI] was purchased from Solvionic (France). All materials were used without further purification.

### Materials fabrication

MINE fabrication involved combining a urethane group–based polymer, synthesized through a one-pot polycondensation process, with a MIL ([Emim][FeCl_4_]). Specifically, 4 mmol polyTHF, 4 mmol DMG, and glycerol (2 mmol for low cross-linking, 5 mmol for medium cross-linking, and 7 mmol for high cross-linking) were dissolved in 14 ml of acetone in a glass bottle under magnetic stirring. Subsequently, IPDI (11 mmol for low cross-linking, 15.5 mmol for medium cross-linking, and 18.5 mmol for high cross-linking) was added, and the mixture was stirred at 500 to 700 rpm at 50°C for 20 hours. After pouring into a PTFE (polytetrafluoroethylene) petri dish, the homogeneous mixture was heated at 90°C for 2 hours. The solvent was then evaporated under a vacuum for 2 to 5 min. The final polycondensation reaction occurred at 80°C for 20 hours, followed by drying under a vacuum at 75°C for 10 hours to obtain dried prepolymers.

These dried prepolymers (also called polyurethane elastomer, PUE) with varying cross-linking degrees were then redissolved in acetone using a heated oil bath at 58°C. MILs were added to the prepolymer solution at different weight ratios. After achieving a well-dispersed mixture using a planetary centrifugal mixer (Kakuhunter, SK-300511) at 2000 rpm for 20 min, the solution was poured into a glass petri dish and dried at RT to form the final MINE films.

The fabrication process for ionoelastomers with [Bmim][FeCl_4_] and [Emim][TFSI] was identical, except for the substitution of the ionic liquids. Urethane-based polymers were obtained by heating the prepolymers at 75°C for 20 to 30 hours under a vacuum to complete the reaction. Fe_3_O_4_- and NdFeB-based composites were prepared by incorporating iron oxide nanopowders and NdFeB microparticles, respectively, as substitutes for MILs in control experiments.

### Materials characterization

A field-emission scanning electron microscope (Hitachi S-4300) and an optical microscope (KSGAOPIN 680V) were used to observe the morphology of the MINEs. Their element-component mapping was conducted through energy disperse spectroscopy (Oxford Instruments). For ultraviolet-visible transmittance measurement, samples were prepared differently: 120-μm-thick films were directly coated onto glass slides, while samples containing various ionic liquids were held in standard quartz cuvettes with a 10-mm path length. FTIR-ATR was performed using an Agilent Cary 660 spectrometer in the range of 400 to 4000 cm^−1^. XRD analysis was conducted using an x-ray diffractometer (LabX XRD-600). TGA was performed using a TA Instruments TGA Q500 under nitrogen protection at a 15°C/min heating rate from 30° to 750°C. Differential scanning calorimetry (DSC) measurements were performed using a TA Instruments DSC 25 at a 10°C/min heating rate from −30° to 100°C. Gel permeation chromatography analysis was performed using a Waters Alliance e2695 HPLC system that was equipped with Waters Styragel HR3 and HR4 columns (4.6 mm by 300 mm) and a Waters 2414 refractive index detector. XPS (Thermo Fisher Scientific Theta Probe) was performed with monochromatic Al Kα x-rays (1486.6 eV) and a pass energy of 40 eV. The etching process was carried out using Ar at 3 keV and 2 μA with a 4 mm by 4 mm raster, resulting in a calibrated etch rate on SiO_2_ to be approximately 3.4 nm/min.

### Magnetic, mechanical, and electrical measurements

Magnetization dynamics measurements of the MINE were conducted under a high vacuum using a Quantum Design MPMS3 SQUID magnetometer. A varying magnetic field from −70 to 70 kOe was applied at an ambient temperature of 300 K, while an ultralow field of 0.5 kOe was exerted on the samples during a temperature sweep from 2 to 300 K. Mechanical tensile tests were performed using a Zwick/Roell Z2.5 instrument. Unless otherwise noted, tensile and cyclic experiments were conducted at a strain rate of 1 mm/s under ambient conditions. Each sample for testing complied with the type V tensile bar specimen with a 3.18-mm width and a 7.62-mm gauge length based on the American Society for Testing and Materials standard D638. Ionic conductivities of the prepared samples were quantitatively computed from the impedance plots measured by an LCR meter (Keysight, E4980A) with a frequency range from 20 to 2 MHz. A digital multimeter (Keithley, DMM7510) was used to measure the resistance changes of the samples while characterizing the strain sensors.

As for the time-lapse self-healing observation, the prepared sample was cut with a sharp knife and then healed under an optical microscope. The electromechanical performance after self-healing was tested by cutting the sample into two pieces and then recontacting the fresh-cut surfaces. The mechanical self-healing efficiency (%) is defined as the ratio of the self-healed elongation to the initial elongation. The detailed quantitative descriptions of resilience (%) and elastic recovery (%) in this work can be found in note S5.

### Hyperelastic constitutive model (Ogden model)

The formulation of Ogden strain energy potential given by ABAQUS software is as follows

$$U=\sum_{i=1}^{N}\frac{2\mu_{i}}{\alpha_{i}^{2}}\left(\lambda_{1}^{-\alpha_{i}}+\lambda_{2}^{-\alpha_{i}}+\lambda_{3}^{-\alpha_{i}}-3\right)+\sum_{i=1}^{N}\frac{1}{\mathrm{Di}}{\left(J_{\text{el}}-1\right)}^{2i}$$ (1)

where $\lambda_{2}=\lambda_{3}={\lambda_{1}}^{-\frac{1}{2}}$ for uniaxial tension. In this work, we set order *N* = 6 for calibration. The value of constant terms $\mu_{i}$ and $\alpha_{i}$ can be obtained by fitting the Ogden model with experimental data in ABAQUS commercial software.

To evaluate the goodness of fit, the coefficients of determination $R^{2}$ are calculated using Eq. 2

$$R^{2}=1-\frac{\text{Sum}\;\text{squared regression}\;\left(\text{SSR}\right)}{\text{Total}\;\text{sum}\;\text{of squares}\;\left(\text{SST}\right)}=1-\frac{\sum {\left(y_{i}-{\hat{y}}_{i}\right)}^{2}}{\sum {\left(y_{i}-\bar{y}\right)}^{2}}$$ (2)

where $y_{i}$ is the test value, ${\hat{y}}_{i}$ is the model fit value, and $\bar{y}$ is the average value of $y_{i}$. Young’s modulus *E* was calculated from linear calibration of the stress-strain curve at a strain range between 1 and 5% as the slope of the calibrated curve.

### DFT simulations

First-principles calculations were performed using the Vienna ab initio simulation package based on the DFT framework. The exchange-correlation function was treated within the generalized gradient approximation using the Perdew-Burke-Ernzerhof functional. The projector augmented wave method was used to describe interactions between valence electrons and ionic cores. A plane-wave basis set with a cutoff energy of 400 eV was used to expand the wave functions, and a 1 by 1 by 1 k-mesh was used to sample the Brillouin zone. The convergence criteria for the energy and the forces on each atom were set to 1 × 10^−4^ to 10^−6^ eV and 0.08 eV/Å, respectively. The magnitude of an electric field was set to 0.1 V/Å, and its direction was along the direction of the third lattice vector (IDIPOL = 3; DIPOL = 0.5 0.5 0.5).

### Noncontact magneto-iono-switch

An ac power supply of 5 V with 100 Hz was applied to the MINE4 magneto-iono-switch. On the basis of the whole circuit design shown in fig. S31, Arduino pins were used to measure voltages across resistors (from R1a to R4a), as well as the ground voltage.

### Low voltage–driven touch panel

A power supply (Agilent, 33210A) was used to supply an alternating voltage of 1 V and 100 Hz to the touch panel. Aluminum foils were used as terminal electrodes of the 1D strain panel based on MINE4 (20 mm by 48 mm by 0.8 mm) and the 2D touch panel based on MINE3 (40 mm by 56 mm by 0.6 mm). The terminal electrodes were then connected to a general-purpose multifunctional data acquisition device (DAQ USB-6221). LabVIEW VI was used to read data from the DAQ, process and filter data (an initial 10 s was used to establish an average value to filter out touch/nontouch data), record data, and communicate data for calibration and mapping to Python scripts. The Python calibration script used the scikit-image Python package to estimate a piecewise affine transformation between raw data and the desired coordinate system. The Python mapping script used the transformation matrix to map raw data from LabVIEW VI to the coordinate system and draw images on a computer screen. The Python pool game used mapped data from a 2D touch panel as mouse input to the position of a pool cue in the game and used mapped data from a 1D strain panel as the pool cue force. The Arduino script used touch information from the mapped data to control the pattern showing on an LCD. MINE4 (20 mm by 5 mm by 0.6 mm) was used as a magneto-iono-switch to drive the display by an external magnet with a magnetic field of 400 mT.

### Soft magneto-iono-carrier system for cargo delivery

An LCR meter (Keysight, E4980A) was used to measure the resistance changes of the MINE4 magneto-iono-muscle during stretching-releasing cycles. The data from the LCR meter were communicated for calibration and mapping to a Python script. Three separate Arduino scripts were used to control the sliding rail, servomotor, light sensor, and LED (fig. S35). The soft magneto-iono-carrier was a hollow square box made of MINEs with dimensions of 30 mm by 30 mm by 25 mm and a wall thickness of around 2.5 mm, fabricated using a layer-by-layer technique. The sides of the box were made of MINE3, while its top and bottom were made of MINE1 for optical transmission and MINE4 for magnetic attraction, respectively.

## Supplementary Materials

### The PDF file includes:

Figs. S1 to S45 Legend for table S1 Tables S2 to S5 Notes S1 to S6 Legends for movies S1 to S7 References
