# Supplementary material for: Self-healing and hyperelastic magneto-iono-elastomers through molecular confinement of magnetic anions
Source: Sci Adv. 2025 Jan 1;11(1):eadq7441. doi: 10.1126/sciadv.adq7441 (PMC11691642; doi:10.1126/sciadv.adq7441)
Supplement: Supplementary file 1 — Figs. S1 to S45 Legend for table S1 Tables S2 to S5 Notes S1 to S6 Legends for movies S1 to S7 References [file sciadv.adq7441_sm.pdf]

Science Advances
AAAS

Supplementary Materials for

Self-healing and hyperelastic magneto-iono-elastomers through molecular confinement of magnetic anions

Xuan Zhang *et al.*

Corresponding author: Yu Jun Tan, yujun.tan@nus.edu.sg

Sci. Adv. **11**, eadq7441 (2025)
DOI: 10.1126/sciadv.adq7441

The PDF file includes:

Figs. S1 to S45
Legend for table S1
Tables S2 to S5
Notes S1 to S6
Legends for movies S1 to S7
References

Other Supplementary Material for this manuscript includes the following:

Table S1
Movies S1 to S7

Supplementary Figures

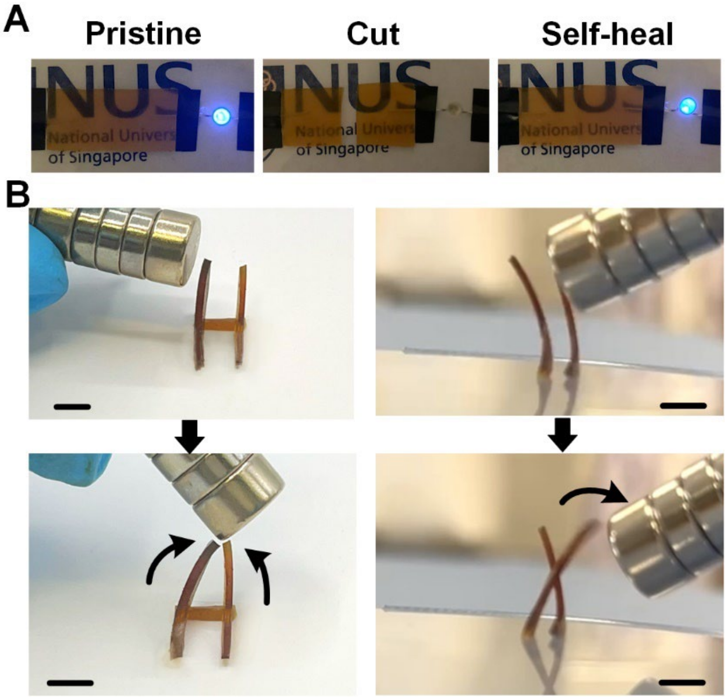

Fig. S1. **Optical photographs of ionic conductive and magneto-responsive MINEs at room temperature.** (**A**) MINE is conductive and can close a circuit to illuminate a blue LED when connected to an AC power source. Its conductivity remained stable after self-healing. (**B**) Driven by an external magnet, MINE can morph its pattern from the letter “H” into “A” and from the Latin number “II” into “X”. Scale bar: 5 mm.

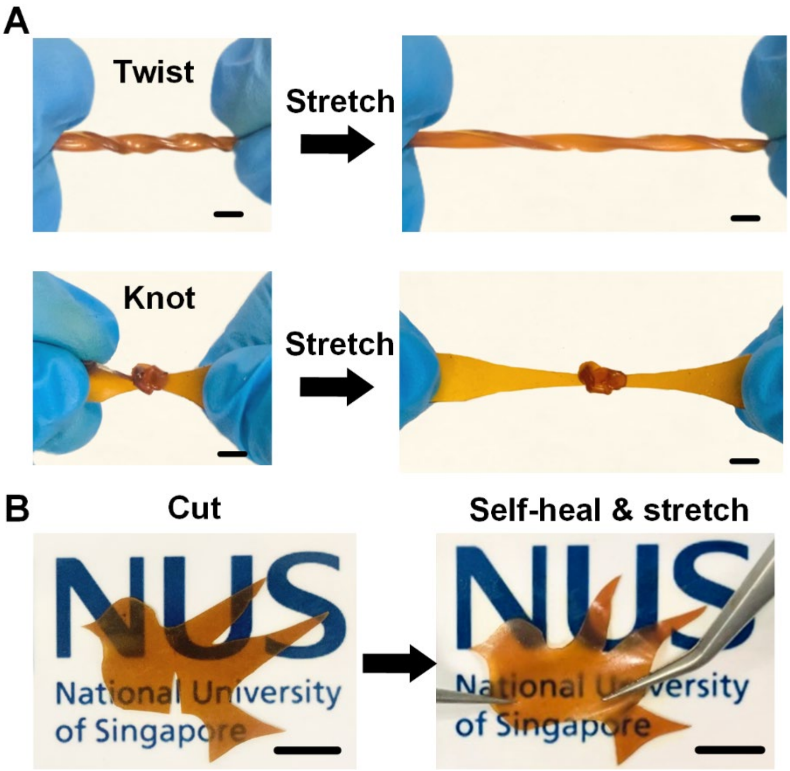

Fig. S2. **Optical photographs of standalone and resilient MINEs at room temperature.** (**A**) MINE could be twisted and tied into a knot and then stretched, showcasing its superior mechanical flexibility and stretchability. Scale bar: 5 mm. (**B**) self-healing performance of the MINE bird at 50 ℃. The reconnected and self-healed cut damage on the bird remained stretchable. Scale bar: 1 cm.

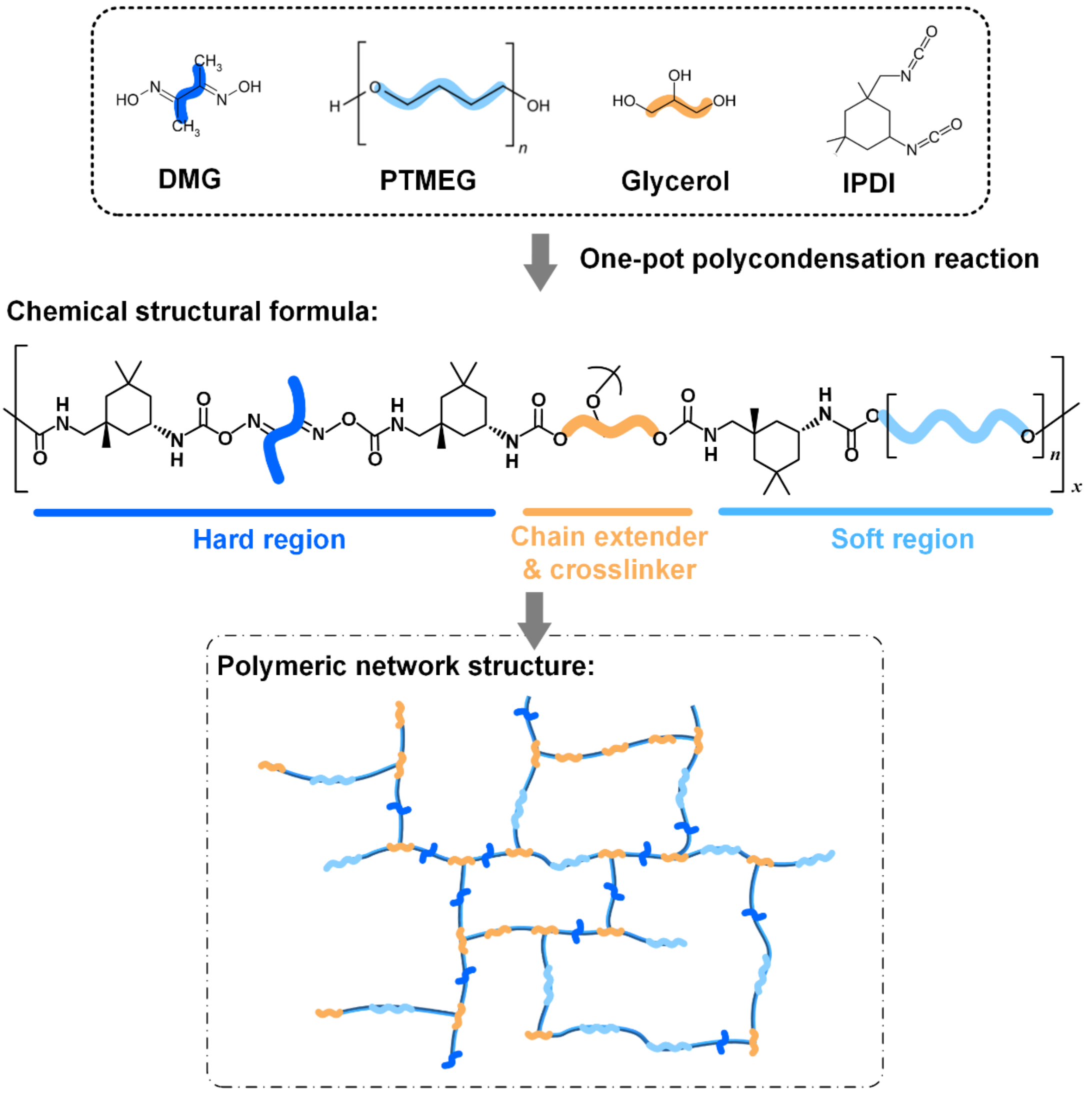

Fig. S3. Network structure of the synthesized elastomer matrix rich in multiple urethane groups.

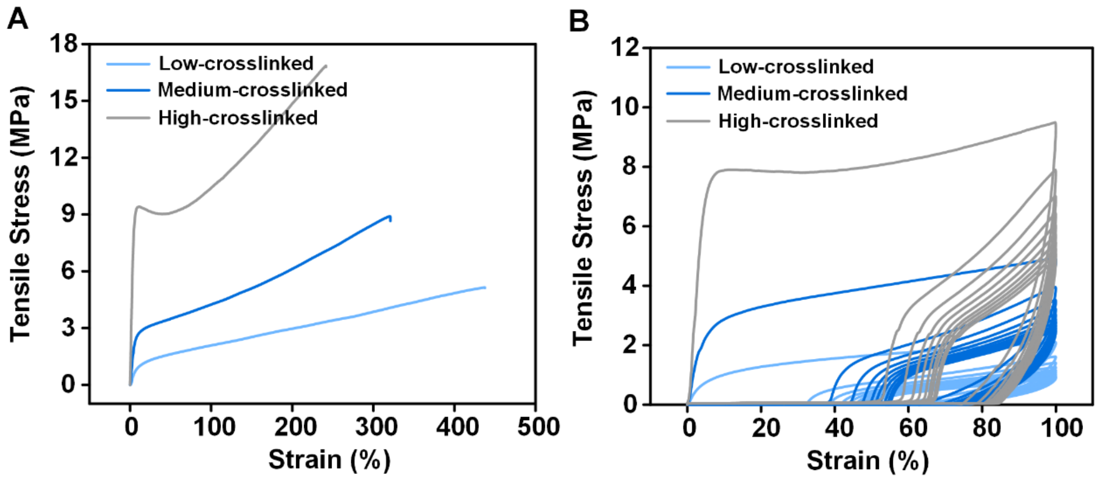

Fig. S4. **Tailoring the mechanical performance of the urethane group-based polymer using glycerol as a crosslinker.** (**A**) Stress-strain curves of polymers with different crosslinking degrees. (**B**) Corresponding cyclic loading-unloading curves of the same polymers. The stiffness of the polymer increases with the degree of crosslinking. Polymers with a higher crosslinking exhibit larger hysteresis loops and reduced elasticity due to restricted chain movement.

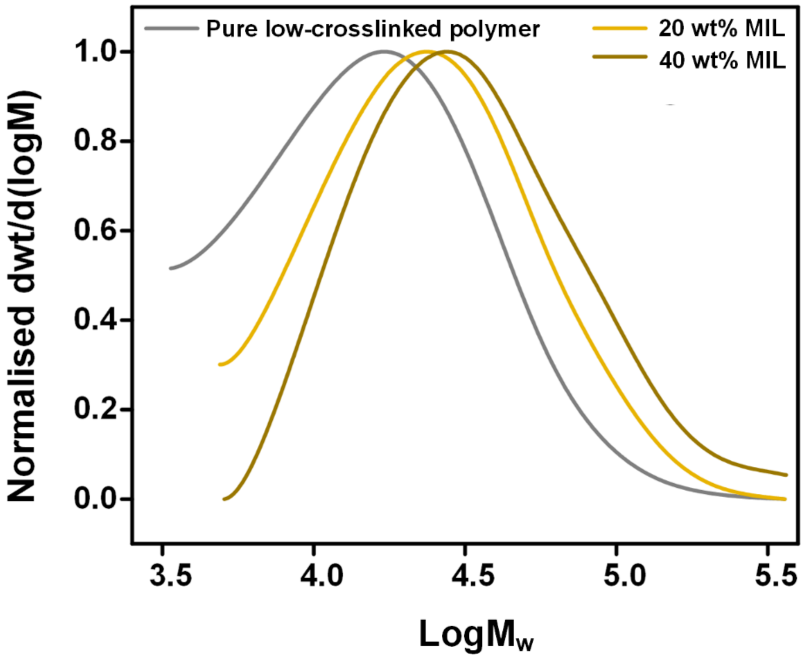

Fig. S5. **The GPC calibration curve of molecular weight for the polymer and MINEs containing different [Emim][$FeCl_4$] contents.** The y-axis refers to the amount of a polymer with a certain molecular weight, x-axis presents the logarithm of the molecular weight corresponding to the slice retention time.

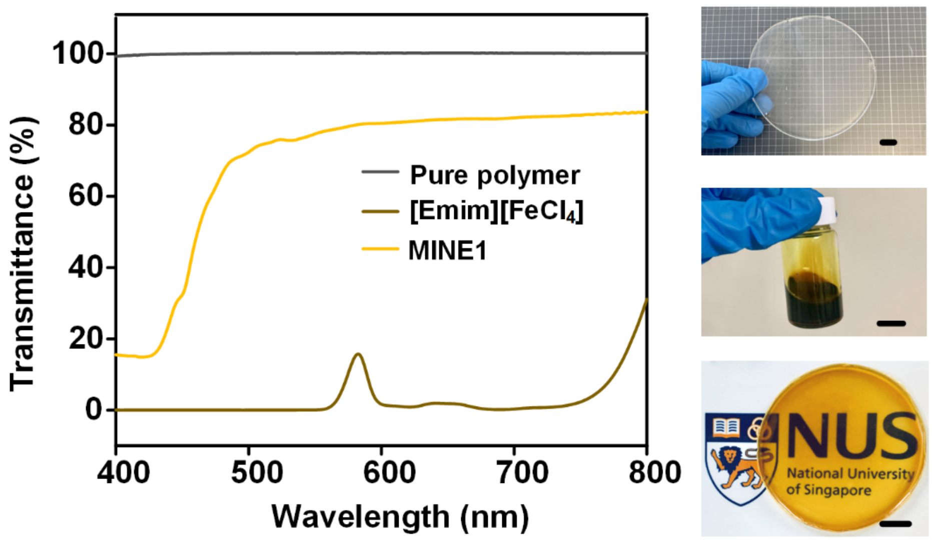

Fig. S6. Optical transparency characterization of MINE. Photographs of a transparent urethane group-based polymer film, brown [Emim][$FeCl_4$] liquid, and a yellowish transparent MINE1 (scale bar: 1 cm), as well as the transmittance spectrum of the three samples.

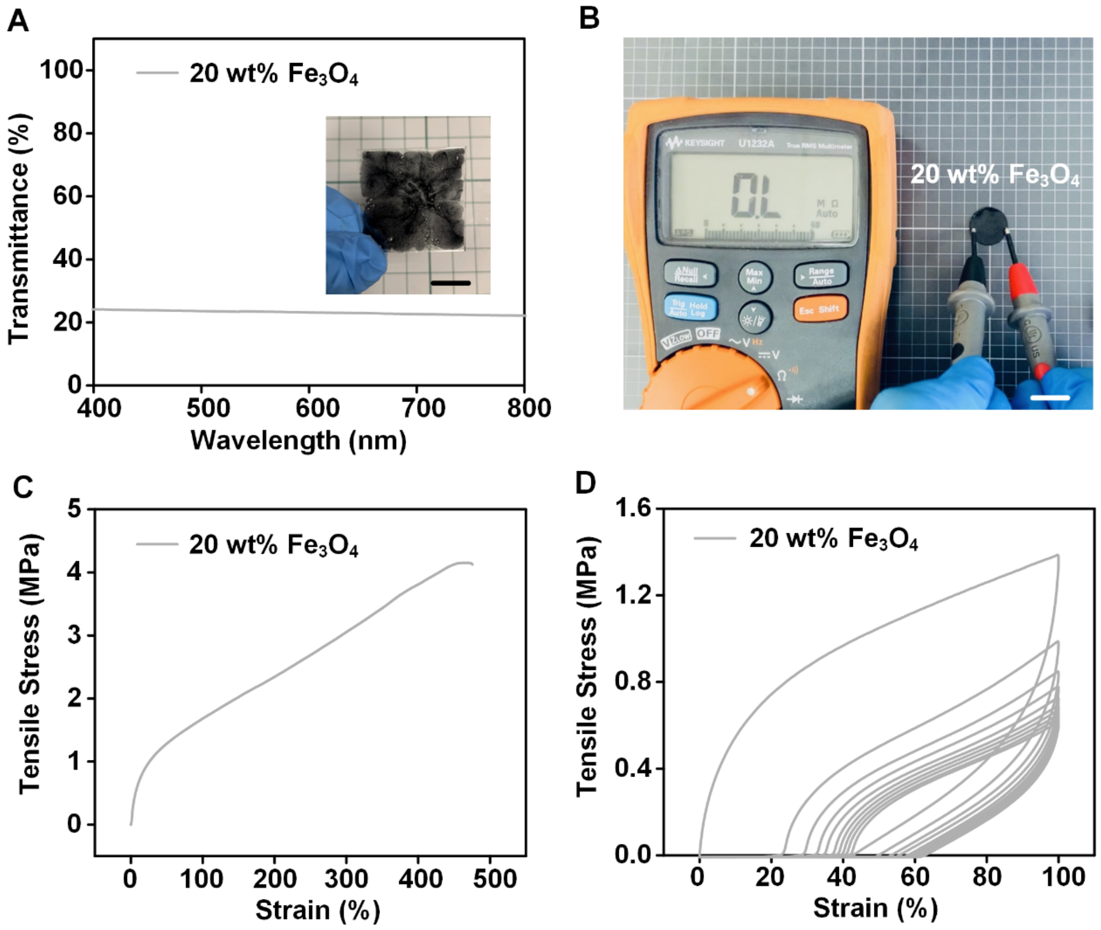

Fig. S7. Characterization of ferromagnetic composites (20 wt% Fe_3O_4 nanoparticles in the low-crosslinked polymer matrix rich in urethane groups) a control material. (**A**) Low transparency of the composite in the visible range (400-800 nm) despite the low Fe_3O_4 concentration, as shown by the transmittance spectrum. The inset image reveals an uneven distribution of ferromagnetic nanoparticles in the composite. (**B**) Photograph of the electrically insulating composite (scale bar: 1 cm). (**C**) Strain-stress curve and (**D**) cyclic loading-unloading curve, respectively, demonstrating the negligible influence of Fe_3O_4 nanoparticles on the mechanical performance of the urethane group-based polymer.

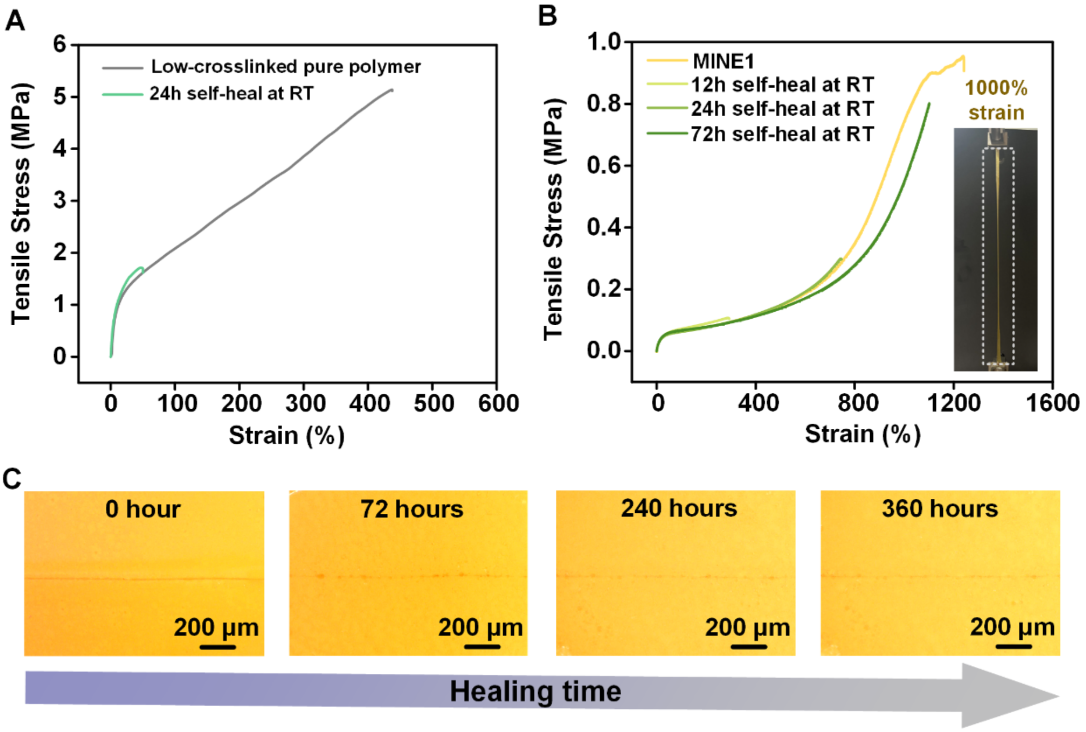

Fig. S8. Mechanical properties and self-healing behavior of MINE1 at room temperature (RT). Stress-strain curves of the pristine and healed states for both (**A**) polymer and (**B**) MINE1. The addition of [Emim][$FeCl_4$] as a plasticizer significantly facilitates the flexibility and self-healing of MINE1. The inset shows a photograph of MINE1 undergoing a 1000% strain. (**C**) Optical microscopy images of the damaged interface in MINE1 following self-healing at RT for 0, 72, 240, and 360 hours. The interface was formed by cutting the MINE1 into two pieces and then allowed to reattach.

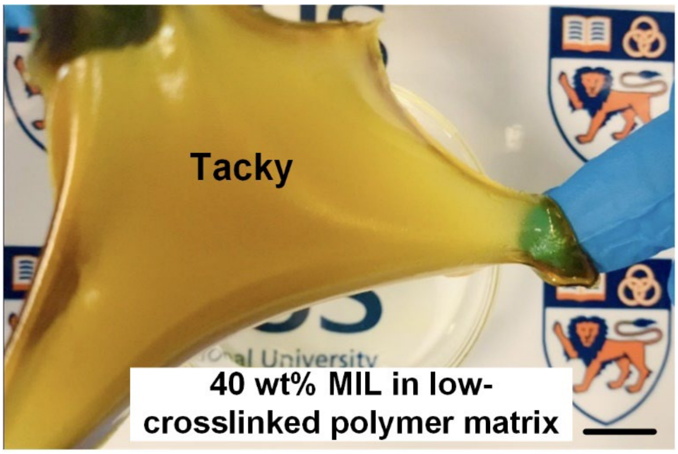

Fig. S9. **Optical photograph of the low-crosslinked polymer with 40 wt% MIL.** The material was tacky and not free-standing (scale bar: 1 cm).

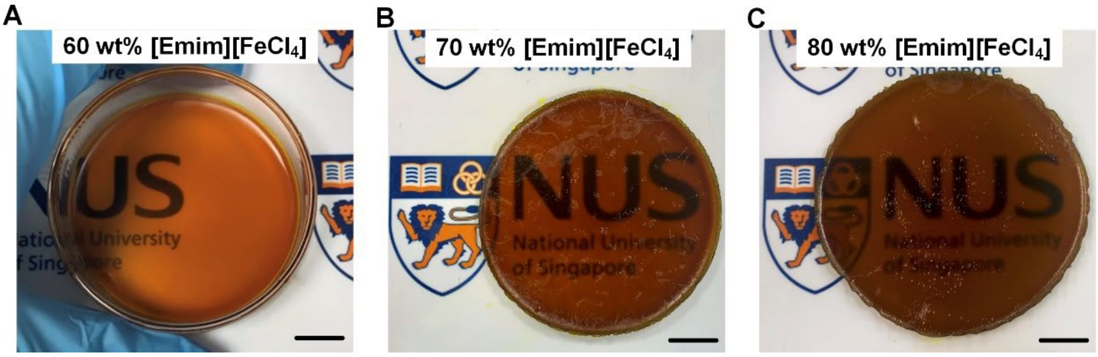

Fig. S10. **Optical photographs of various MINEs with different MIL contents:** (**A**) MINE4, (**B**) MINE5, and (**C**) MINE6. All the samples were ~1 mm in thickness (scale bar: 1 cm).

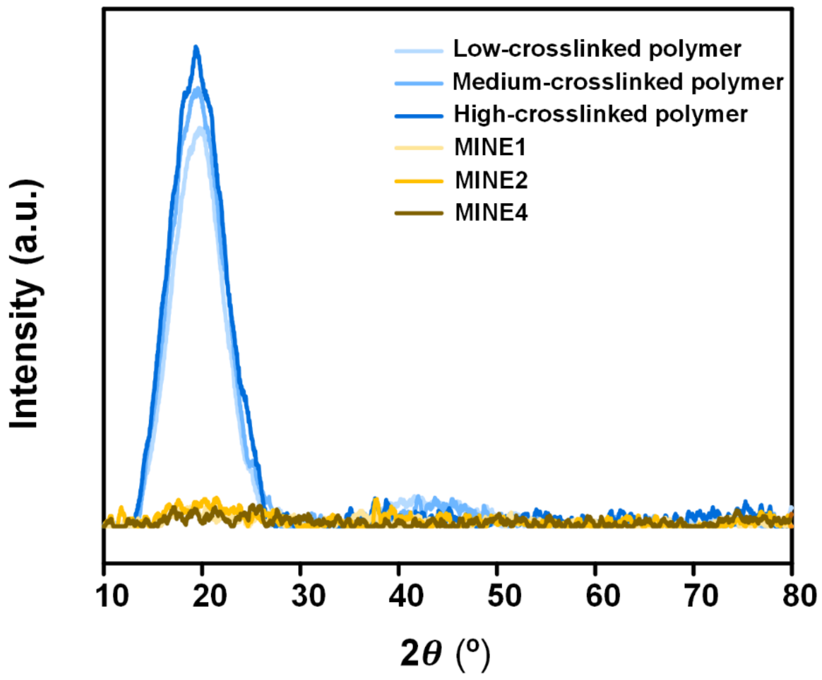

Fig. S11. **XRD characterizations of various pure polymers and MINEs.** All polymers with different crosslinking degrees reveal broad peaks around 20°, indicating the amorphous nature of the polymer chains. These peaks, potentially due to small crystalline domains within the pure polymers (*30*, *31*), were flattened upon the addition of [Emim][$FeCl_4$]. This suggests interactions between the urethane group-based polymer and MIL, leading to a fully amorphous material (*32*).

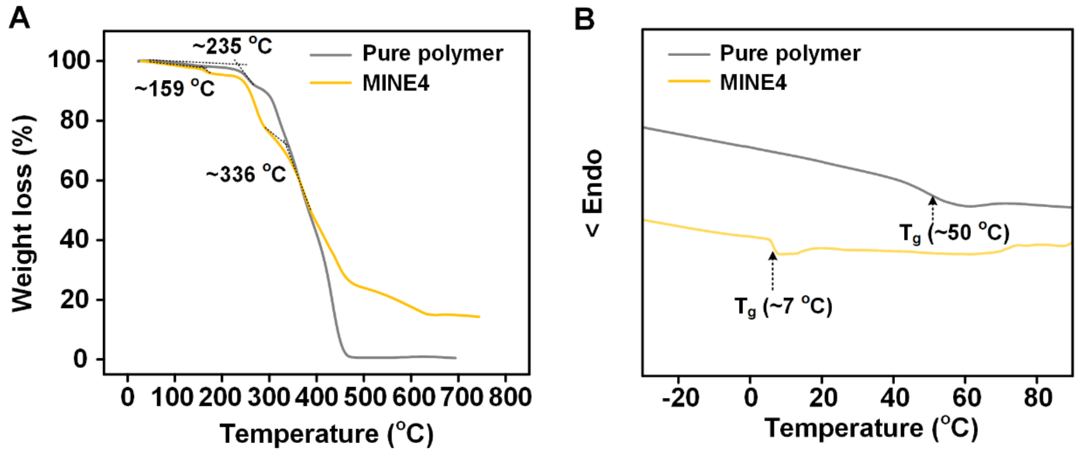

Fig. S12. **Thermal analysis of urethane group-based polymer and MINE.** (**A**) TGA and (**B**) DSC measurements of the polymer and MINE4 containing 60 wt% [Emim][$FeCl_4$] in a nitrogen environment. The pure polymer has a higher initial

decomposition temperature at 235 ℃ as compared to MINE which is at 159 ℃. When the temperature is at 336 ℃, the thermal decomposition of MINE is mainly attributed to [Emim][$FeCl_4$] through dealkylation (*33*, *34*). The plasticization effect by MIL is expected to predominate, rendering the system more thermally sensitive. This is evidenced by the decline in the glass transition temperature (T_g) (*35*, *36*). Moreover, the decrease in the decomposition temperature serves as an indication of the strong electrostatic interaction between the cations and the urethane groups (-NHCOO-) during the thermal degradation process, confirming the presence of ion-dipole interaction within the MINE system (*37*).

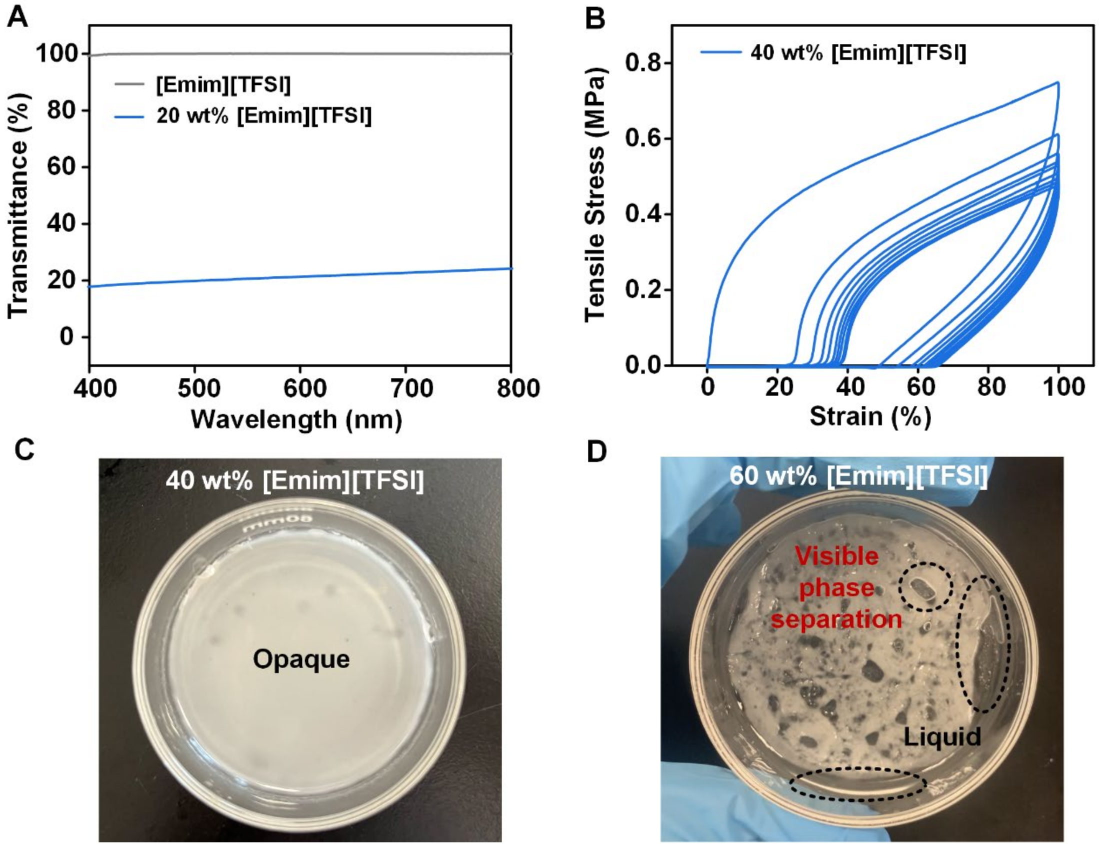

Fig. S13. Characterization of control materials: urethane group-based polymers with [Emim][TFSI]. (**A**) Visible-range (400-800 nm) transmittance spectra of pure [Emim][TFSI] and the low-crosslinked polymer with 20 wt% [Emim][TFSI]. (**B**) Cyclic loading-unloading curves of the control material containing 40 wt% [Emim][TFSI]. (**C**,**D**) Optical photographs of the high-crosslinked polymer with 40 wt% and 60% wt%

[Emim][TFSI], respectively. The control materials exhibit minimal transparency, and the 60 wt% [Emim][TFSI] sample shows visible phase separation evidenced by pockets of viscous liquid.

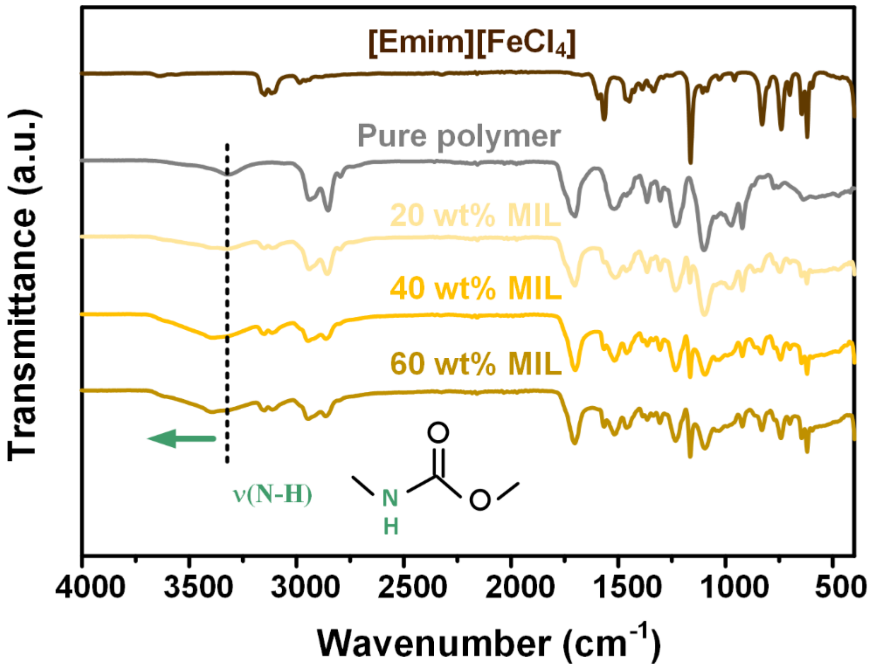

Fig. S14. FTIR-ART spectroscopy of pure urethane group-based polymer, MIL, and MINEs. The aromatic C-H vibration of $[Emim]^+$ was observed in the peaks ranging from 3105-3200 cm^{-1}, at ~1165 cm^{-1} and ~619 cm^{-1}, while the peak at ~830 cm^{-1} for its aliphatic C-H vibration. The transmission bands around 3100-3200 cm^{-1}, 1500-1650 cm^{-1}, and 1100-1200 cm^{-1} belonged to the metal chloride from $[FeCl_4]^-$ (*33*, *34*, *38*), and their peak intensity increased with the MIL mass ratio. MINEs show a distinct peak shift at ~3317 cm^{-1} corresponding to the N-H group in urethane groups. The higher the MIL concentration, the broader the peak and the more apparent the peak shift. Many amide units in PUE chains can act as hydrogen donors, enabling them to form "potential hydrogen bonds (PHBs)" with the Cl atoms from the MIL anions, in which Cl atoms serve as hydrogen acceptors. Besides, the flat regions at 2264 cm^{-1} corresponding to N=C=O in the IPDI monomer were evident that the polymerization reactions had completely taken place.

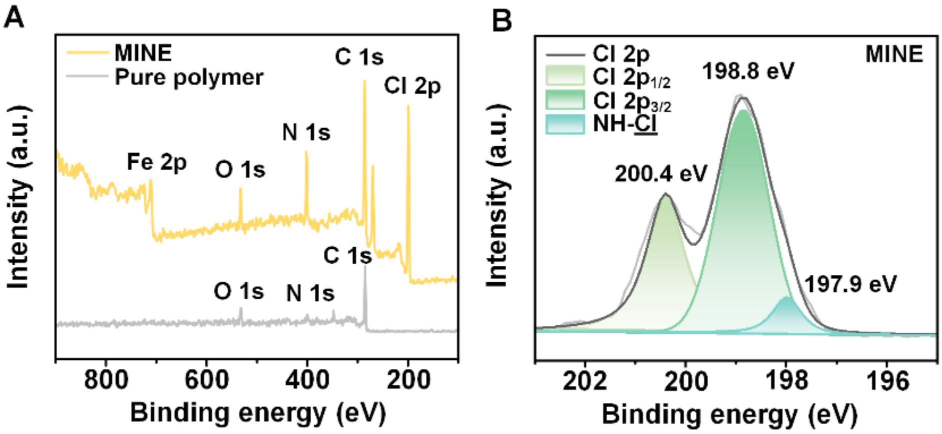

Fig. S15. **X-ray photoelectron spectroscopy of MINE**. (**A**) XPS spectra of urethane group-based polymer and MINE and their corresponding high-resolution spectra: Cl 2p (**B**). The significantly higher peak intensity in MINE as compared to pristine polymer suggests a notable rearrangement of elements and hydrogen bonding from the bulk to the surface sites, facilitating intramolecular and intermolecular interactions. Oximate nitrogen (C=N–O) and amido N (-C(=O) NH-) were confirmed at 400.4 eV (*33*), and 398.9 eV, respectively (*34*, *35*). The incorporation of MIL induces a substantial peak downward shift of 1eV in the amide N (-NH-C=O) peak observed in the spectrum (*15*, *39*). Additionally, the emergence of a new peak at 197.9 eV in the Cl 2p spectrum, attributed to NH-Cl interactions (*40*), further validates the bonding feasibility between urethane groups and Cl atoms from MIL anions (PHBs). Notably, MINE exhibits another peak at 399.4 eV in the N 1s spectrum, which is associated with the N-metal interaction (Fe-N) (*16*). Compared to the bonding of Fe (III) and amide O, the Fe-N $_{amide}$ bonding is stronger (*41*). Furthermore, the peak at a binding energy of 714 eV in the Fe 2p spectrum of MINE further confirms the existence of Fe-N coordination bonds (MCBs), which are absent in pure [Emim][$FeCl_4$] ionic liquids (**Fig. 2D**) (*42*, *43*).

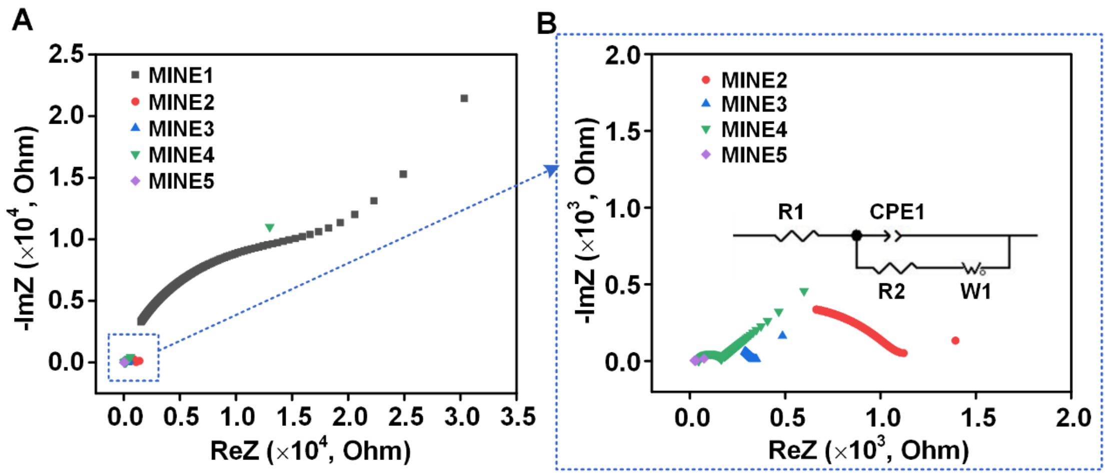

Fig. S16. **The influence of MIL loading on electrical properties of various MINEs.** (**A**) Nyquist plots of various MINEs with different MIL concentrations. ReZ and ImZ are defined as the real and imaginary parts of the impedance (Z), respectively. (**B**) Zoomed-in graph of (A). Inset is the equivalent circuit of the MINEs.

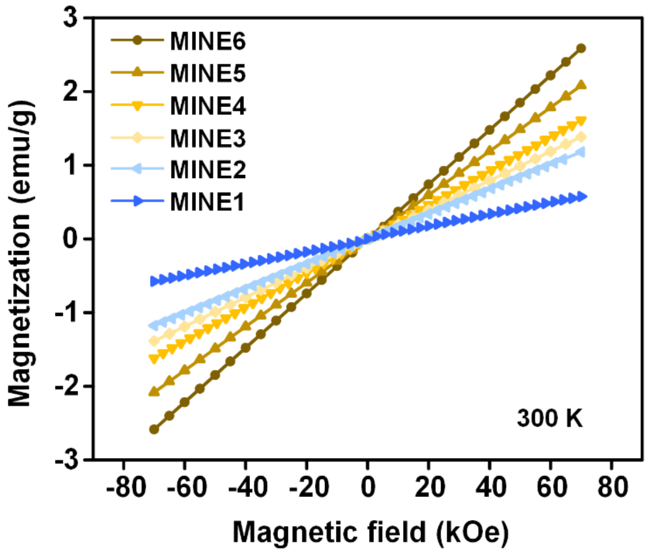

Fig. S17. **Magnetization of various MINEs (20-80 wt% MIL contents) as a function of applied magnetic field at room temperature.**

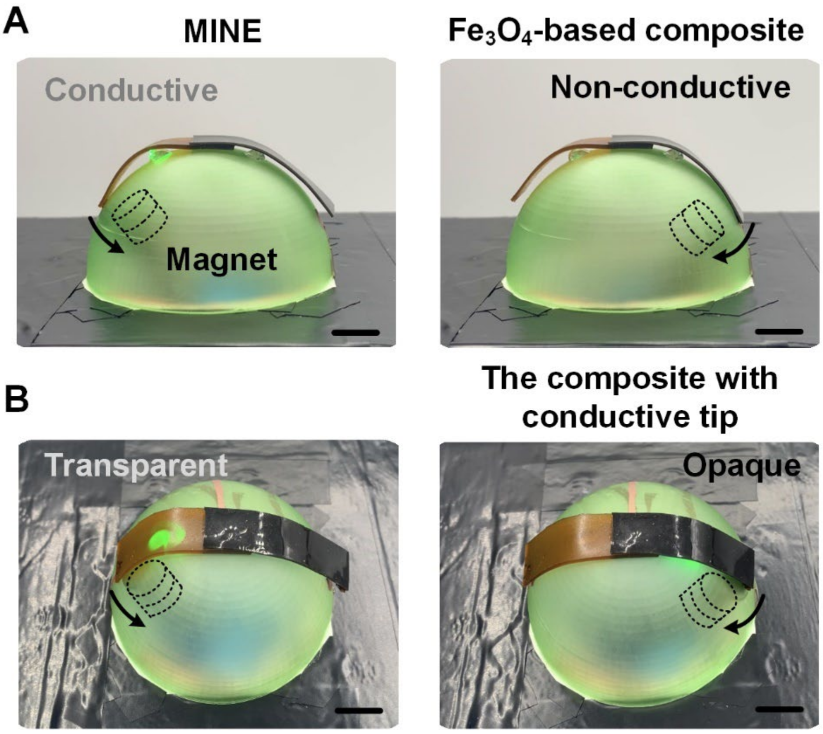

Fig. S18. Photographs demonstrating the multifunctional advantages of MINE compared to a Fe_3O_4-based composite (scale bar: 1 cm). (**A**) The MINE combines an all-in-one superiority in conductivity, magneto-responsiveness, and optical transparency, which is absent in the magnetic particle-filled composite. (**B**) The conductive composite was prepared by attaching a MINE piece to the composite film's tip. For magnetic actuation, a magnet was positioned within the green hemisphere.

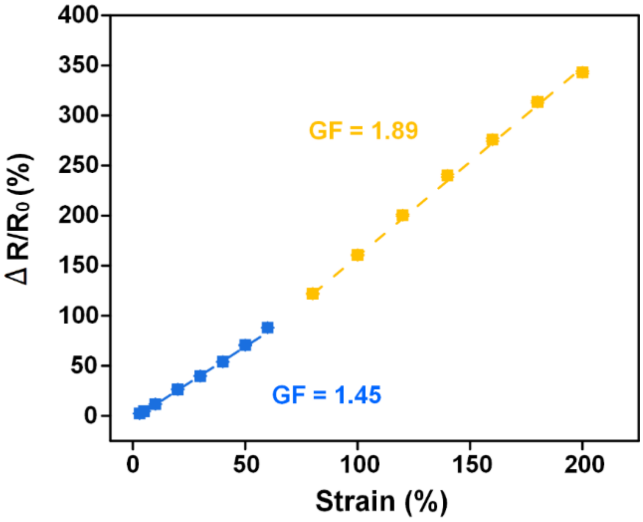

Fig. S19. Relative resistance-strain plot of MINE4. Blue and green dot lines represent the linear fitting in the ranges of 0%-60% and 60%-200% strain, respectively.

Their slopes correspond to different gauge factors (GF) within the two applied strain ranges. The gauge factor (GF), expressed as $\mathrm{GF} = (\Delta\mathrm{R}/\mathrm{R}_0)/\varepsilon$, was used to represent the sensitivity, where $\Delta\mathrm{R}$ and R_0 are the relative resistance change in response to the applied strain ε and the nominal resistance R_0, respectively.

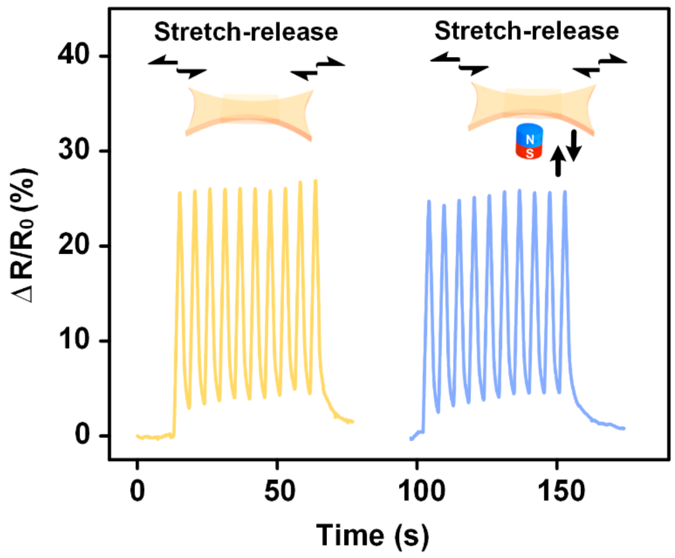

Fig. S20. The influence of a strong magnetic field on the electrical resistance of MINE4 during stretch-release cycles. As an external magnet (magnetic field of 400 mT) was repeatedly moved closer to and further away from the material at random speeds, no discernible interference in its resistance outputs was observed.

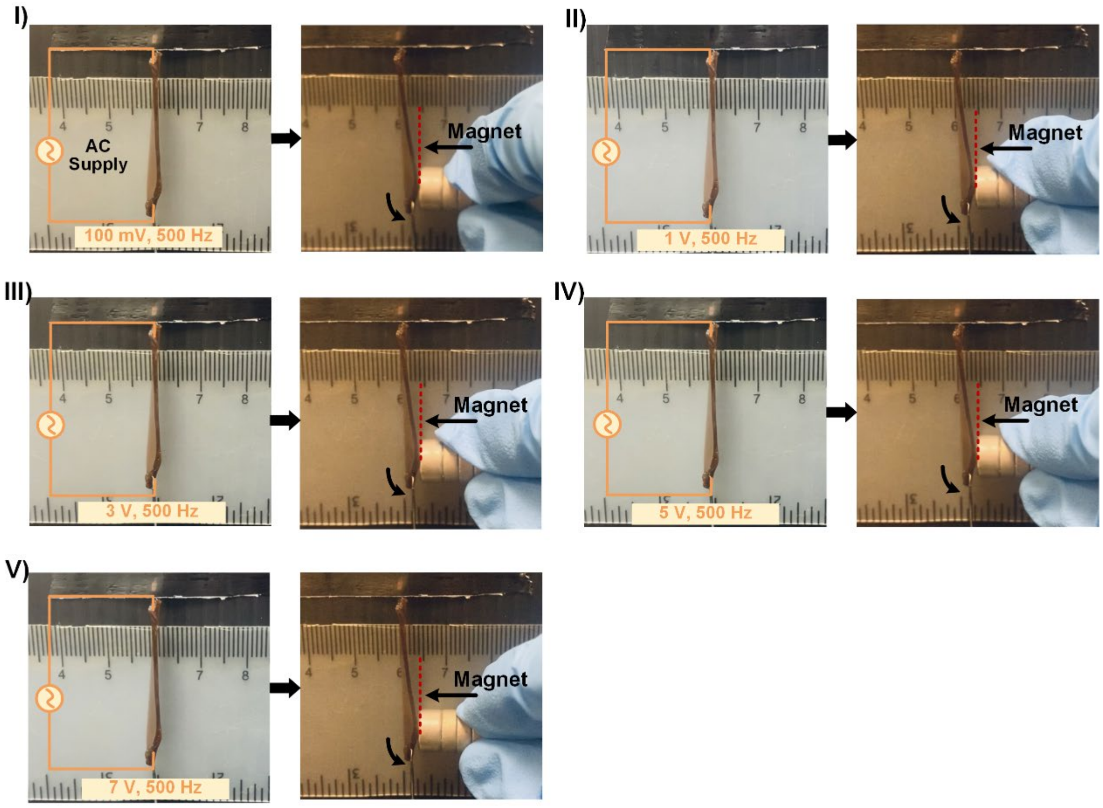

Fig. S21. The influence of AC fields at varying voltages on the magneto-responsiveness of MINE4. For all the cases, MINE4 had the same minimum responsive distance when approached by the same external magnet (magnetic field of 200mT).

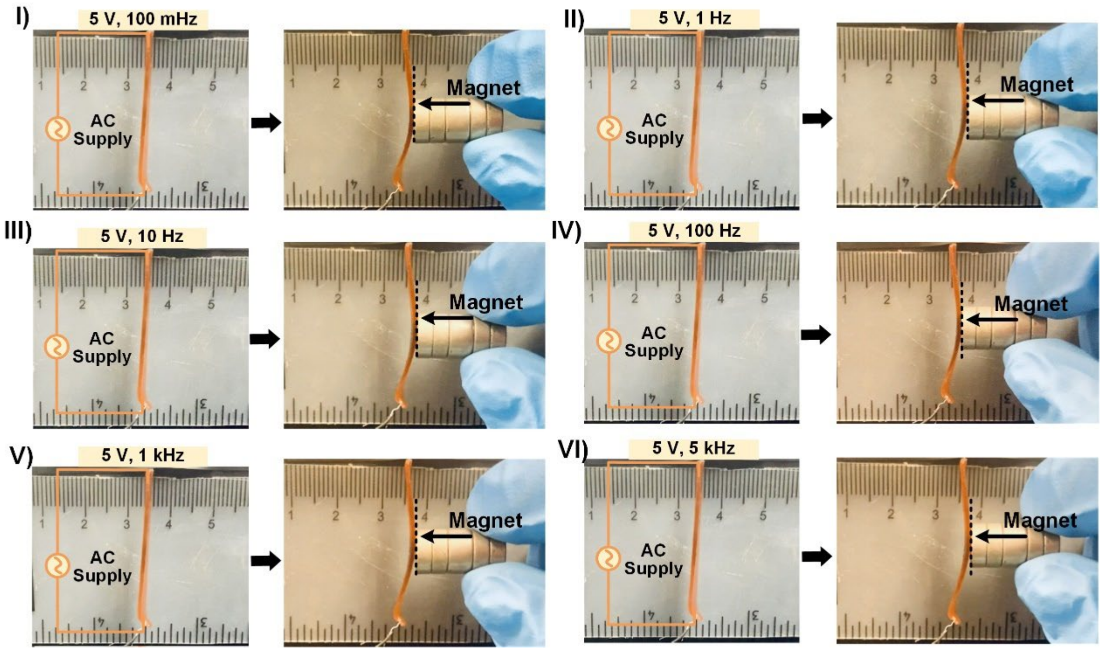

Fig. S22. The influence of AC fields at varying frequencies on the magneto-responsiveness of MINE4. For all the cases, MINE4 had the same minimum responsive distance when approached by the same external magnet (magnetic field of 200 mT).

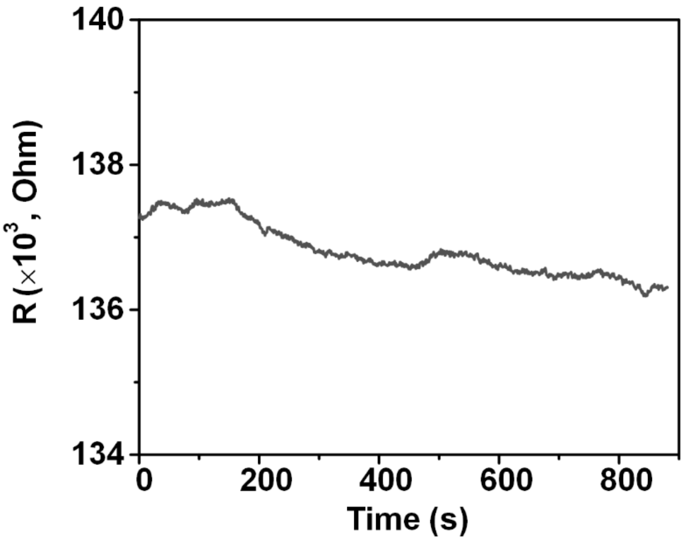

Fig. S23. The influence of resistance outputs in a fixed MINE4 when a strong magnet approached or moved away from it. There was no discernible and regular fluctuation in resistance output when a strong magnet was repeatedly approaching and removing from the MINE4 in the circuit. This indicates that the external magnet had no significant impact on the electrical signal output of MINE4, which is promisingly designed for magnetically actuated sensors.

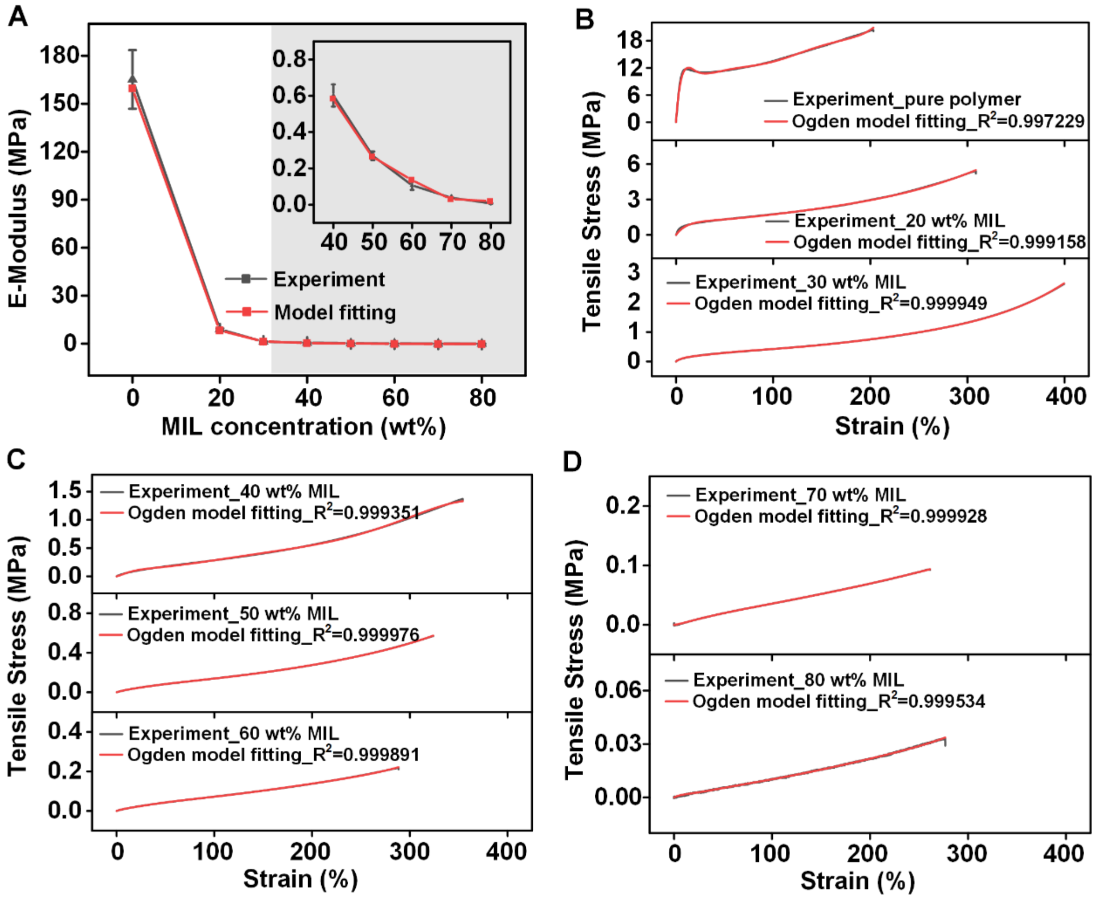

Fig. S24. Young's modulus of MINEs followed by Ogden hyperelastic model. (**A**) E-modulus (Young's modulus) of the high-crosslinked polymer and various MINEs including experimental data and fitting data *via* a hyperelastic constitutive model. For hyperelastic materials, their E-modulus was calculated from linear calibration of the stress-strain curve at a strain range between 1% to 5% as the slope of the calibrated curve. The stress-strain relationship $\sigma_1(\lambda)$ in fitting data can be obtained by calibrating Ogden hyperelastic model in Abaqus software. Error bars indicate standard deviations from three measurements for each point. (**C-D**) The results obtained from fitting the Ogden hyperelastic model for the high-crosslinked polymer and different MINEs demonstrate R^2 values above 0.9.

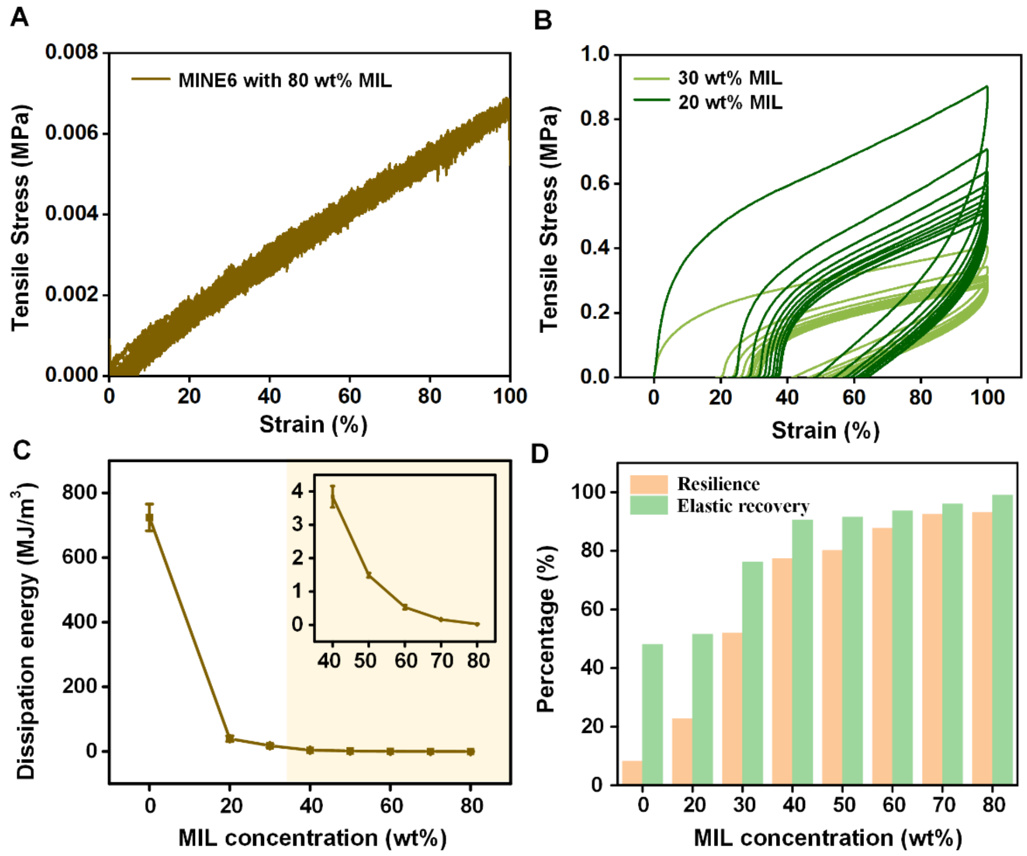

Fig. S25. Mechanical resilience of high-crosslinked polymer and various MINEs. Cyclic loading-unloading curves of different MINEs with (**A**) 80 wt% MIL, and (**B**) 20 wt% & 30 wt% MIL. The nearly linear curves during stretching-releasing cycles indicate minimal energy loss, reflecting superior resilience. (**C**) Dissipation energy comparison for the pure polymer and MINEs. Error bars indicate standard deviations from three measurements for each point. The dissipation energy of the 80 wt% MIL sample is nearly 151 times lower than that of the 40 wt% MIL counterpart and over 2.8×10^4 times lower than that of the pure polymer. (**D**) Resilience and elastic recovery for various MINEs, both showcasing gradual increases with the MIL content.

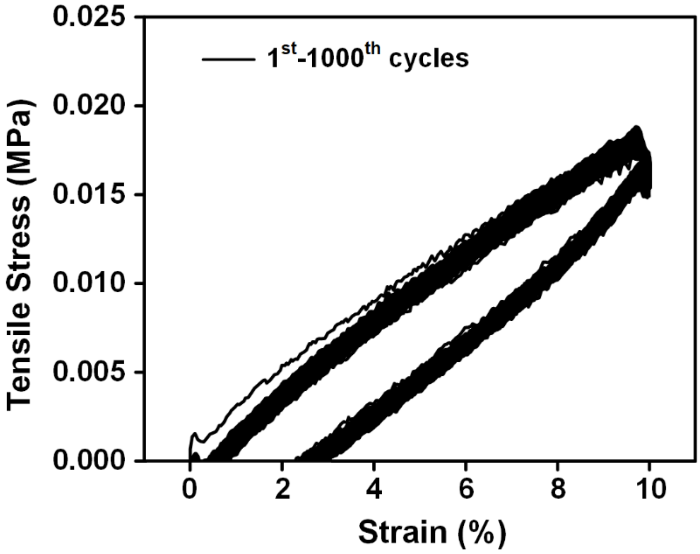

Fig. S26. Cyclic loading-unloading curves of MINE4 at a strain of 10% for a continuous 1000 cycles.

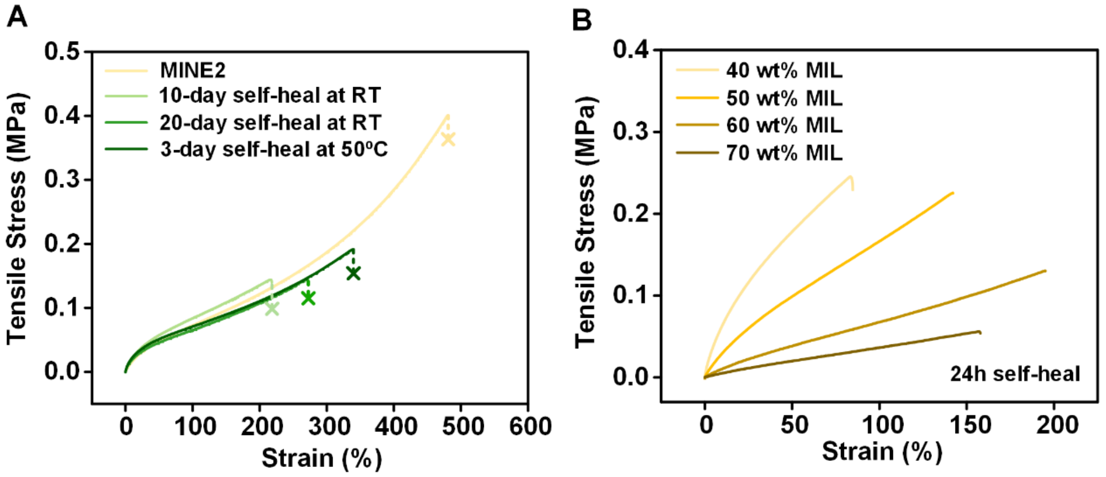

Fig. S27. Self-healing performance of MINEs involving higher crosslinked polymers. (**A**) Self-healing performance of MINE2 under different healing conditions. Heating at 50 ℃ shortened the healing time of the MINE with high crosslinked PUEs. After 3-day self-healing, both materials could recover more than 70% of the initial mechanical performance. (**B**) Stress-strain curves of the high-crosslinked polymer mixed with various MIL concentrations after 24h of accelerated self-healing at 50 ℃.

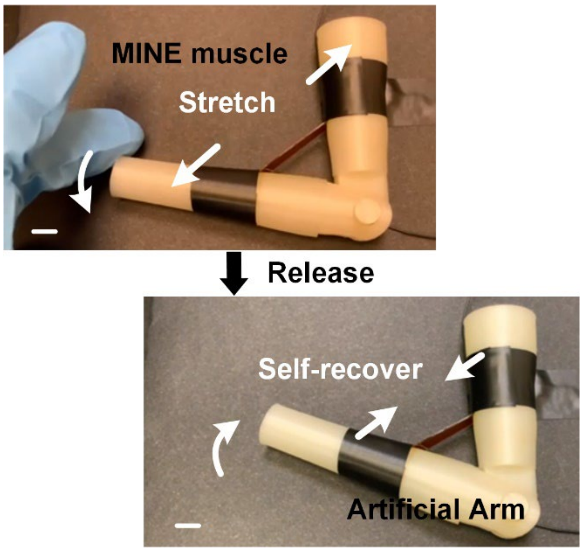

Fig. S28. **Photographs of an artificial magneto-iono-muscle constructed from MINE4, captured during a stretch-release process** (Scale bar: 1 cm). This MINE sensor, boasting both hyperelasticity and ionic conductivity, unlocks exciting possibilities for smart artificial muscles in applications such as electronic skins and soft robotics.

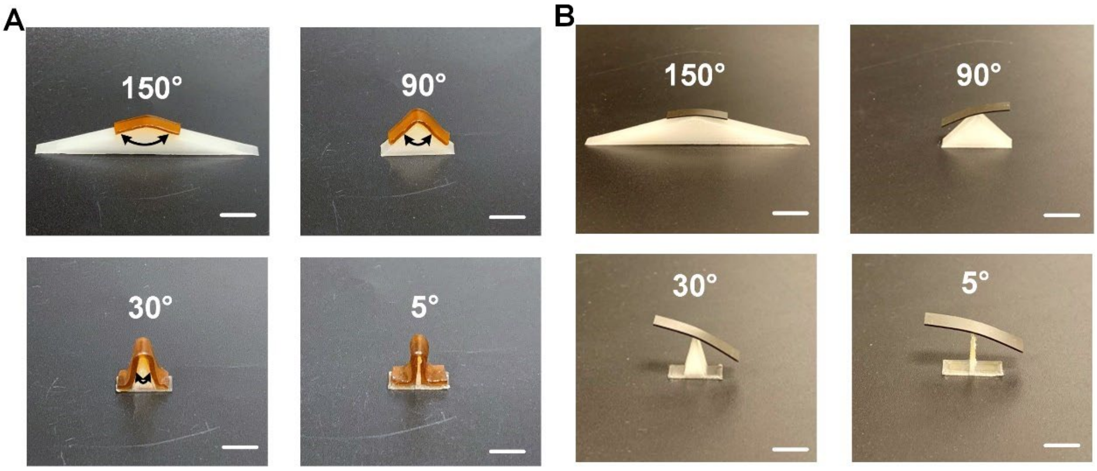

Fig. S29. **Comparison of MINE and ferromagnetic particle-based composite for surface conformality.** (**A**) MINE with excellent conformality on surfaces at various angles. (**B**) NdFeB microparticle-based soft materials struggle to conform to different surfaces, particularly sharp ones. Scale bar: 5 mm

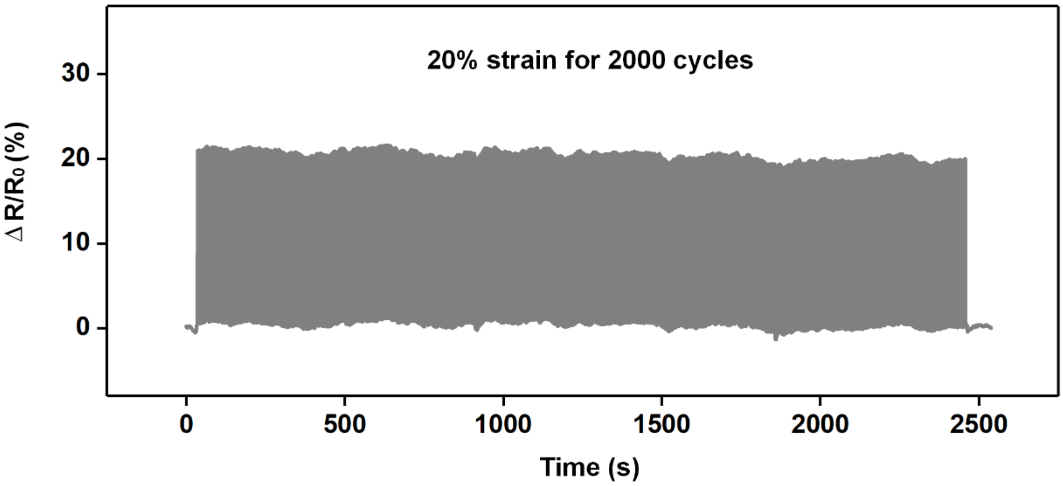

Fig. S30. Resistance changes of MINE4 during 2000 continuous stretch-release cycles at 20% strain, demonstrating long-term electrical reliability.

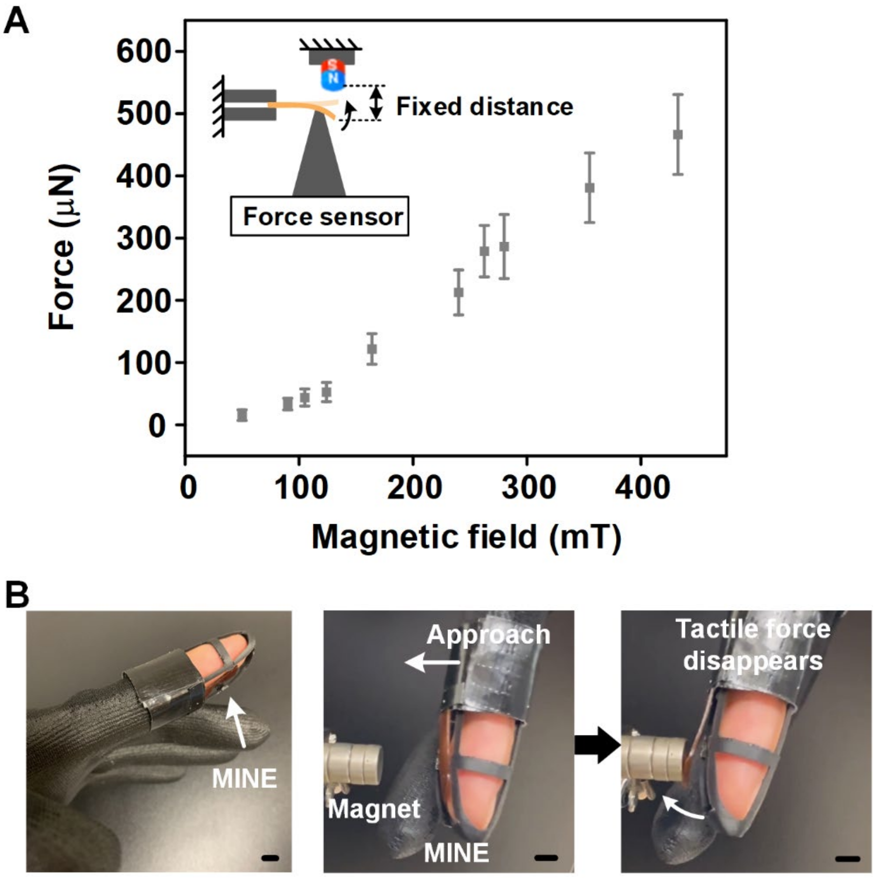

Fig. S31. **Magnetic-actuation characterization of MINE and its potential for integration into smart haptic gloves.** (**A**) Magnetic-actuation force tests of MINE4 at a varying external magnetic field. Scale bar, 5mm. (**B**) Photographs of specifically

designed smart gloves integrated with MINE4 film. Initially, the MINE integrated into a finger of the glove would simply rest against the fingertip skin, providing a tactile sensation (scale bar: 5 mm). Upon approaching a magnet, the MINE could be activated, causing the gentle tactile force on the fingertips to disappear. This change in tactile feedback enables users to directly perceive the existence of an external magnetic field, potentially applying it for enhancing haptic feedback and touchless object manipulation.

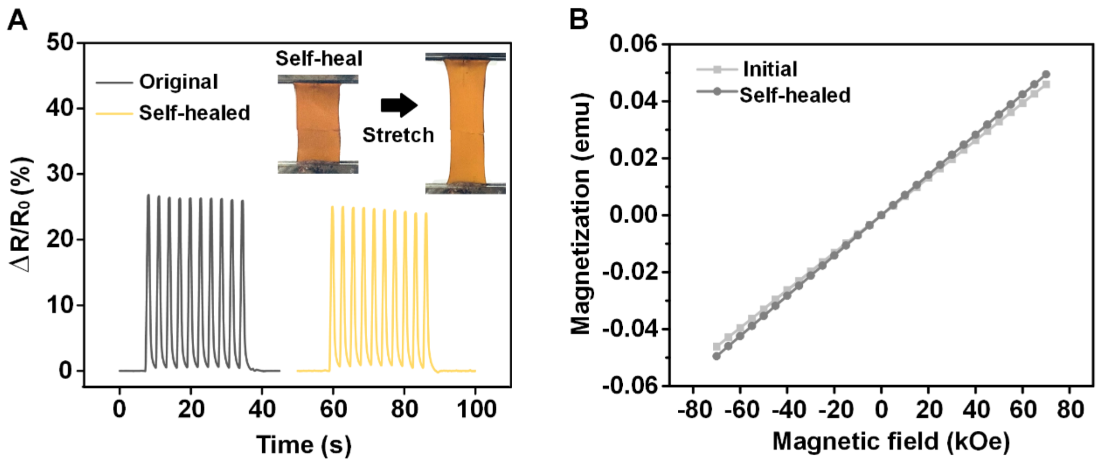

Fig. S32. **The Influence of self-healing on the electrical and magnetic properties of MINE.** (**A**) Resistance changes for the pristine and healed MINE4 at a strain of 50%. Inset: photographs of the self-healed MINE4. (**B**) Magnetization of MINE4 before and after self-healing, measured as a function of applied magnetic field at room temperature.

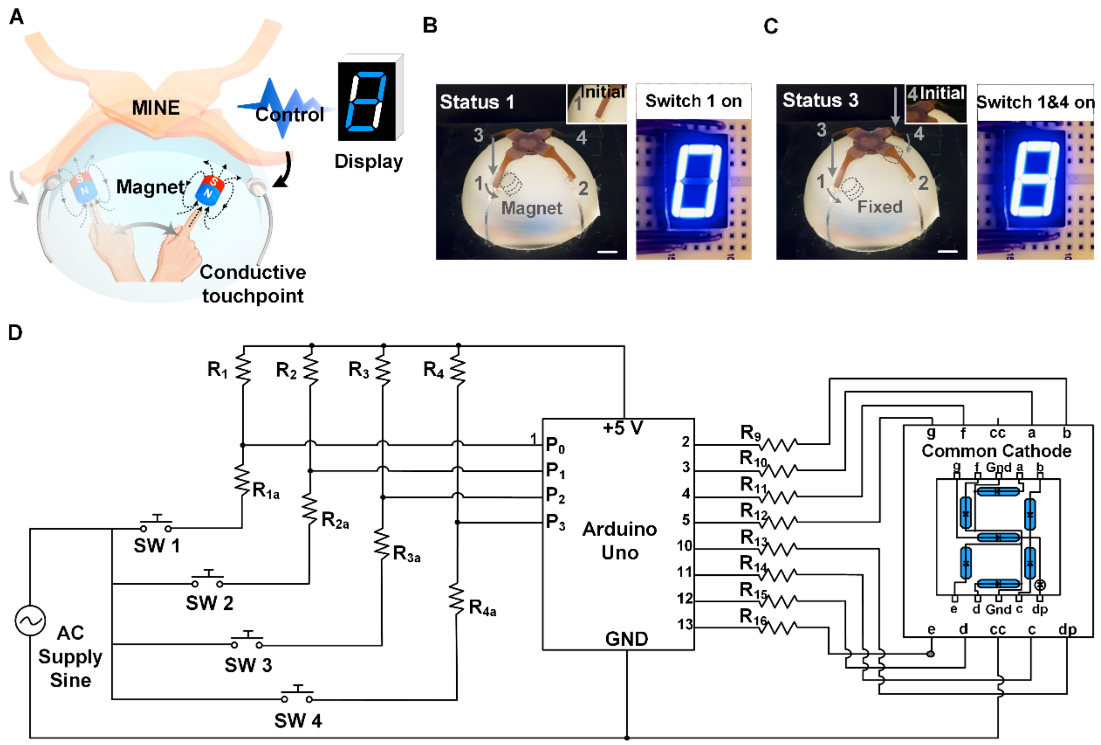

Fig. S33. Demonstration of a contactless magneto-iono-switch system based on MINE. (**A**) Schematic illustration of the system based on MINE4 for seven-segment display control. (**B**) A single-switch actuation mode of the system. Each MINE leg functions as an independent switch, enabling the display of distinct digits through individual control. (**C**) Double-switch actuation mode of the system. By simultaneously activating any two switches, a different designated digit could be displayed. Scale bar: 5 mm. (**D**) An equivalent electrical circuit of the setup. The four switches of the magneto-iono-switch are denoted by SW1, SW2, SW3, and SW4, respectively. A 5V voltage divider was used to condition the signal from an alternative current (AC) power supply to an Arduino Uno for driving the display.

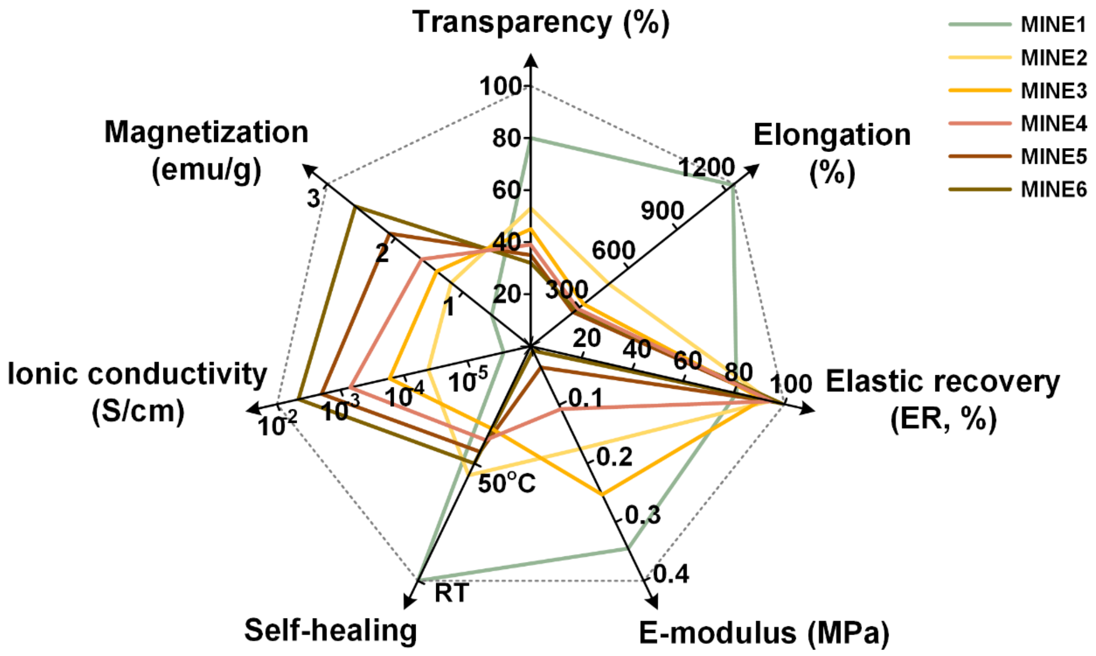

Fig. S34. The individual performance advantages of various MINEs.

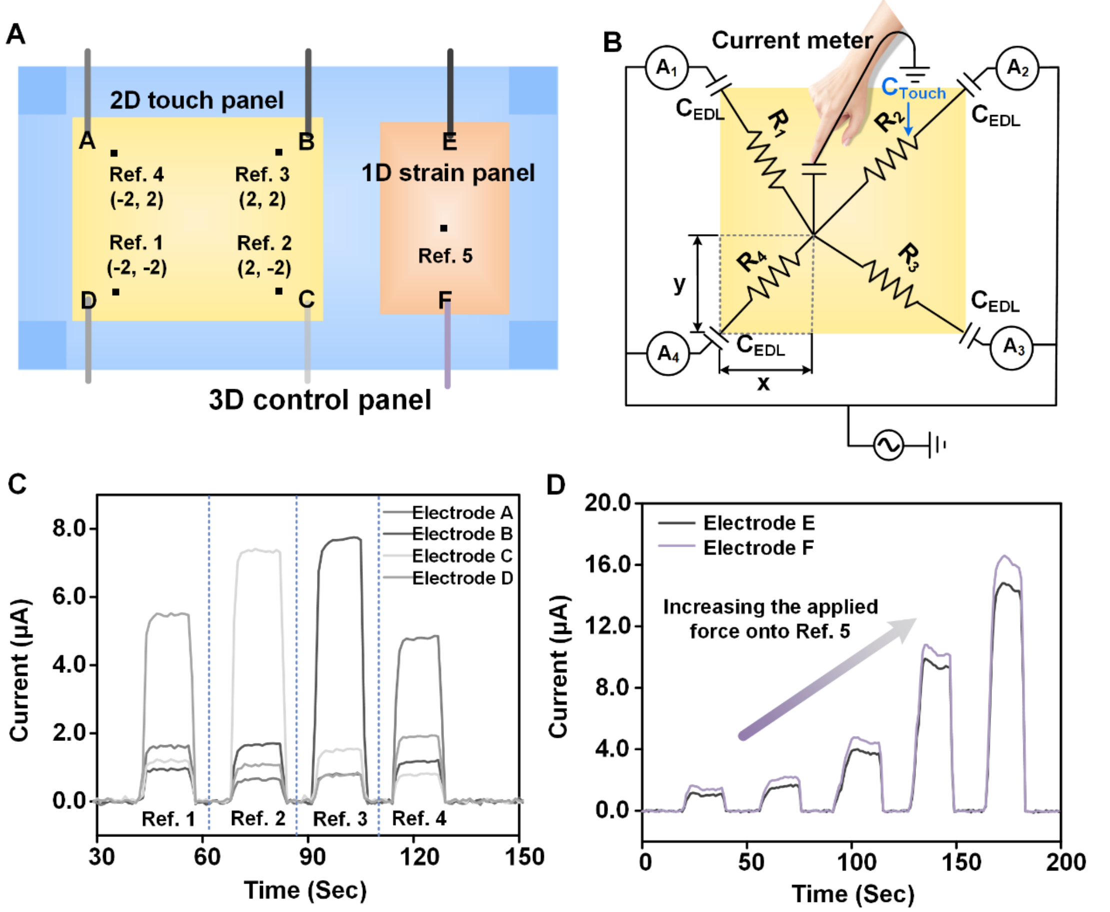

Fig. S35. Calibrations of 2D touch and 1D strain panels in the 3D control panel.

(**A**) Schematic illustrating electrodes (A, B, C, and D) and reference touchpoints (Ref. 1, Ref. 2, Ref. 3, and Ref. 4) of the 2D touch panel for pressure sensing, as well as electrodes (E and F) and reference touchpoint (Ref. 5) of 1D strain panel for strain sensing. In this case, we supplied an AC voltage of 1 V and a frequency of 100 Hz. (**B**) A schematic diagram of MINE3 being used as a pressure-sensing touch panel. A touched position in the surface capacitance system of the ionic elastomer is defined by two normalized distances, x and y. With touching the four Ref. 1, Ref. 2, Ref. 3, and Ref. 4 touchpoints sequentially, the corresponding current outputs are plotted in (**C**). MINE4 was chosen as a strain-sensing touch panel. (**D**) After touching the Ref. 5 point five times with increased force, two electrodes (E and F) measured the current outputs. The current outputs across the two electrodes were then averaged and used to calibrate the 1D strain panel. As a result, the strain panel can be utilized to control the strength of the pool cue in the game.

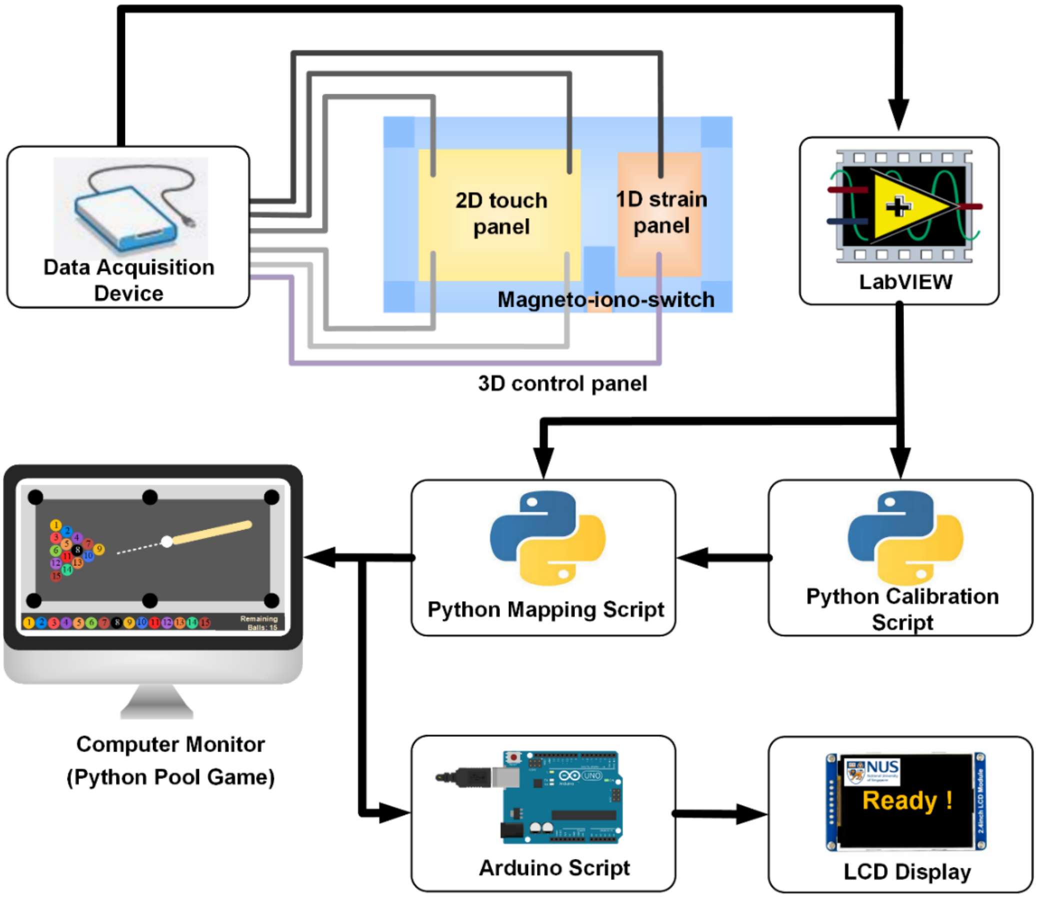

Fig. S36. **Schematic illustration of an ultralow-voltage-driven touch panel system**

applying for 8 Ball Pool game control. 1D and 2D touch panels were connected to a data acquisition device (DAQ), which functioned as an analog-to-digital converter. The data from the DAQ was read by LabVIEW VI, then processed and filtered, as well as communicated for calibration and mapping to Python script. Mapped data was then passed to the Python Pool game and Arduino for execution. Arduino script was used to control the pattern change on the LCD. The magneto-iono-switch in the 3D control panel was used as a turn-on/off button to control the LCD.

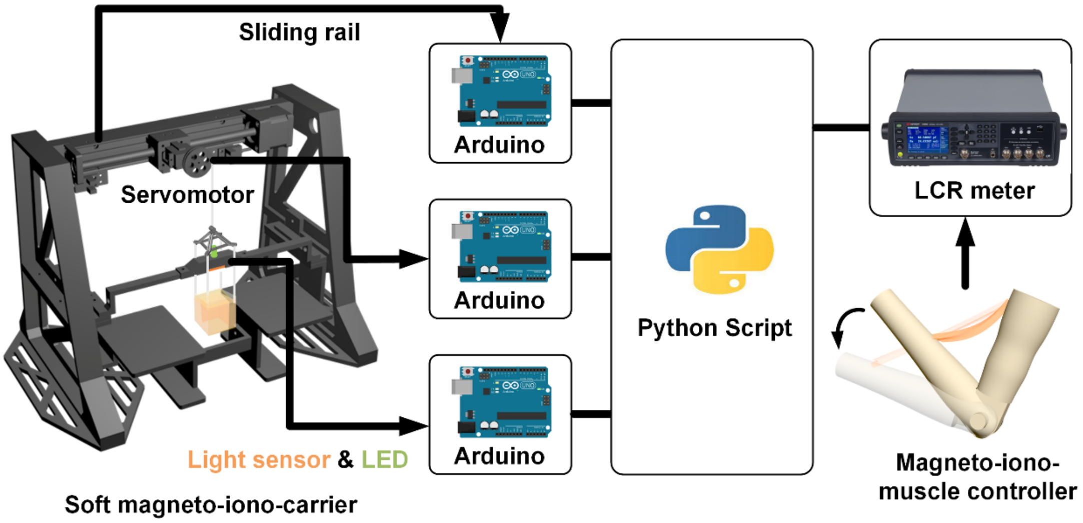

Fig. S37. Schematic illustration of a soft magneto-iono-carrier system controlled by a hyperelastic magneto-iono-muscle for cargo delivery. We selected MINE3 for constructing the robust wall and MINE1 for the transparent top of the carrier, respectively. The hyperelastic MINE4 was designed into the artificial magneto-iono-muscle controller.

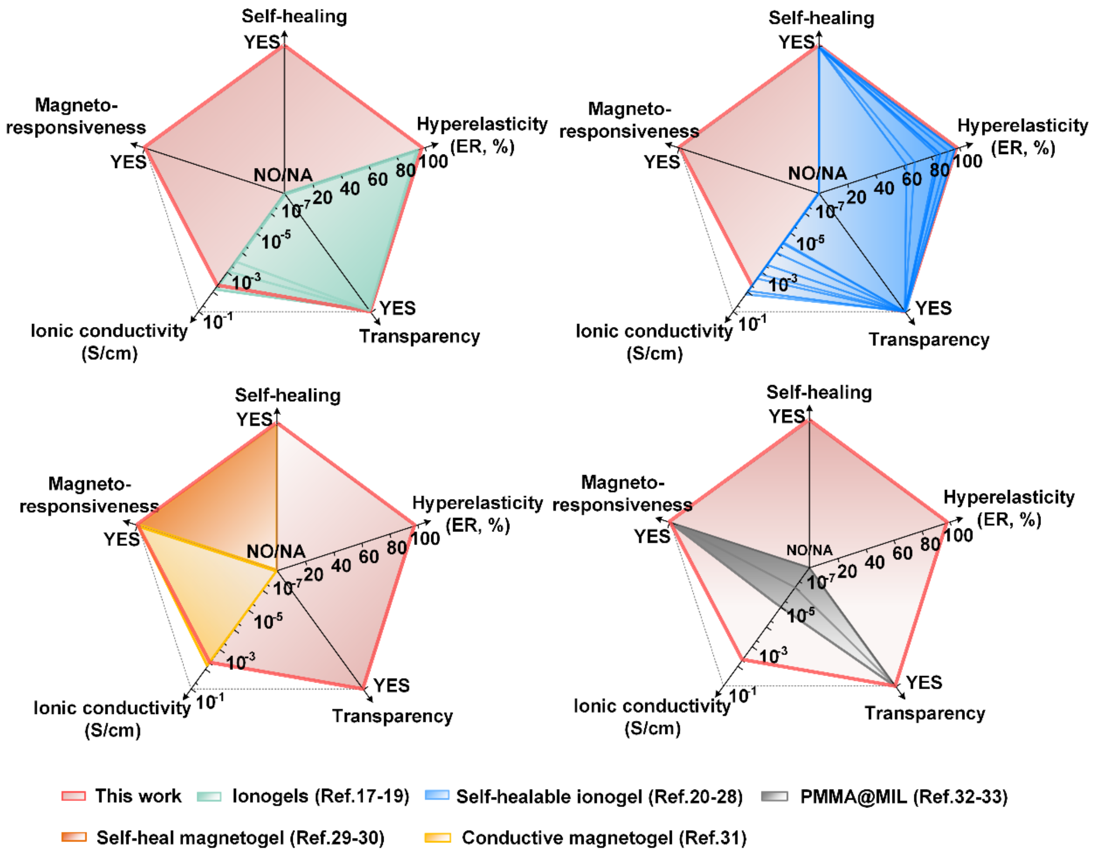

Fig. S38. Comparison between this work and other previous studies (*6*, *7*, *11*, *12*, *18*, *20*, *44-54*).

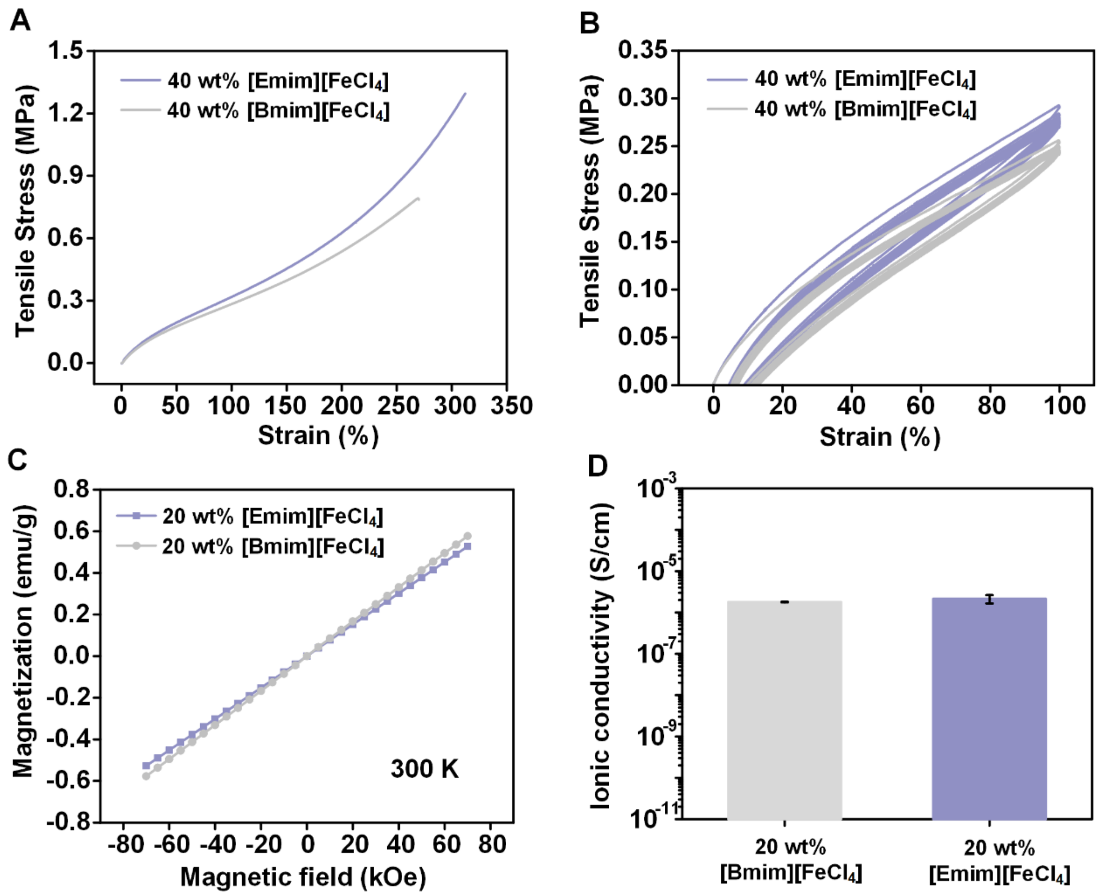

Fig. S39. MINEs with [Bmim][$FeCl_4$]. A magneto-iono-elastomer with PUE and a paramagnetic ionic liquid, [Bmim][$FeCl_4$] was synthesized. (**A**) Strain-stress curves and (**B**) cyclic tensile curves of the two MINEs at the same stretch ratio. Due to them having the same [$FeCl_4$] anion in the two MILs, the MINEs exhibited similar stretchability and elasticity. The longer alkyl side chains often give rise to higher material viscosity, leading to the relatively lower E-modulus of the ionic elastomers containing [Bmim][$FeCl_4$]. (**C**) Their magnetization and (**D**) ionic-conductivity results showed that both MINEs had very similar electrical and magnetic responses.

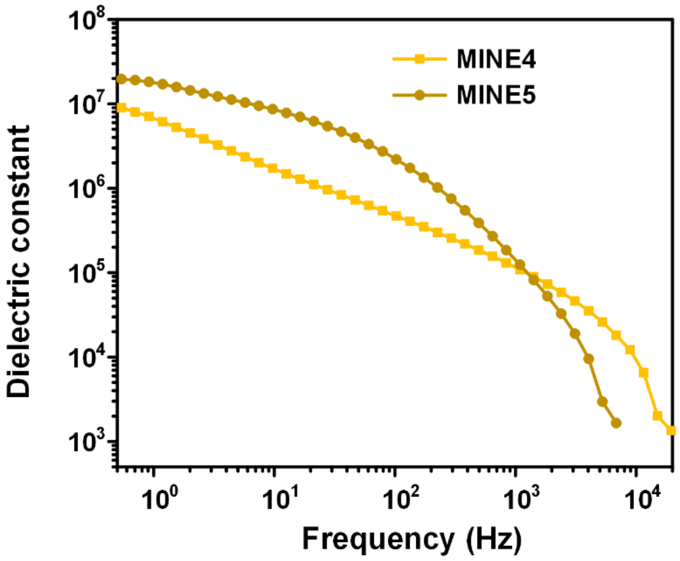

Fig. S40. **Dielectric constant (k) measurement of MINEs.** MINE is an ultrahigh-k dielectric material.

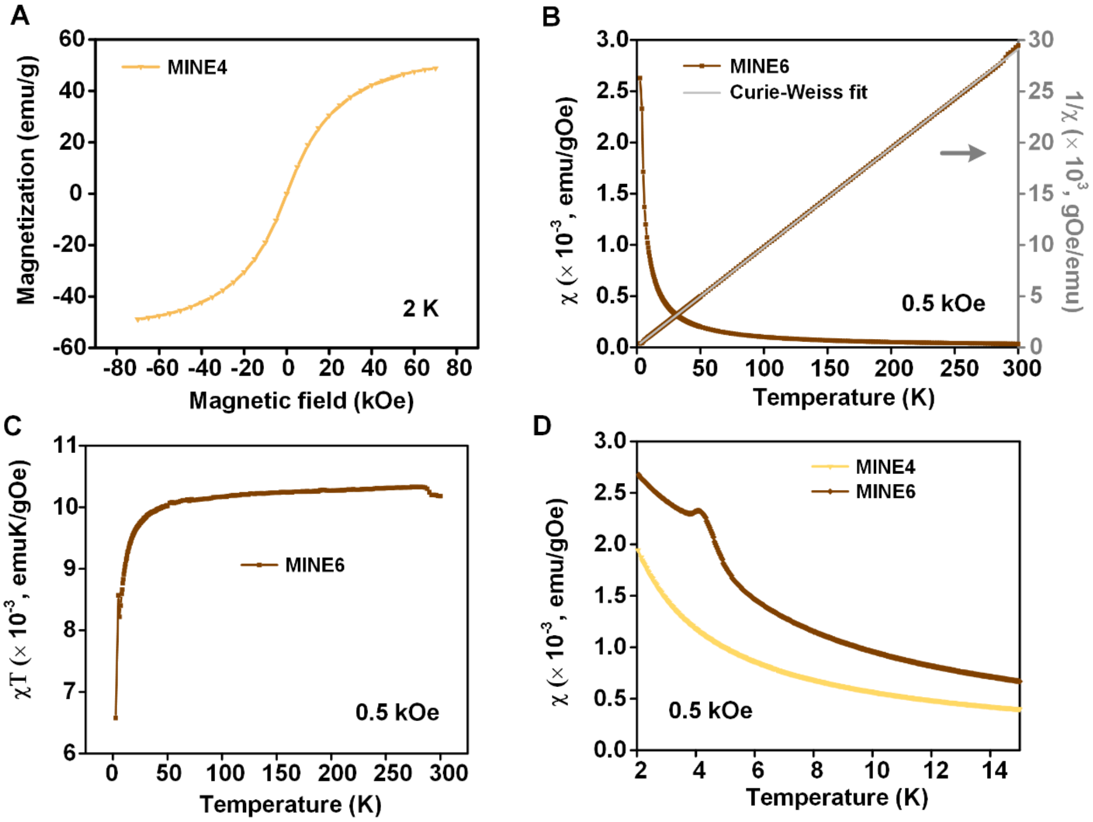

Fig. S41. Other magnetic properties of MINEs. (**A**) Magnetization of MINE4 (60 wt% [Emim][$FeCl_4$]) as a function of an applied magnetic field at a temperature of 2 K. (**B**) Temperature dependence of mass susceptibility (χ and $1/\chi$) for MINE6 (80 wt%

[Emim][$FeCl_4$]) was tested under 0.5 kOe. According to the Curie-Weiss law, the fitted line had a Curie-Weiss temperature (θ_{CW}) of -1.56 K, indicating antiferromagnetic interactions when the molecular field was aligned with the external field. (**C**) Temperature dependence of the effective magnetic moment (χT), having a Curie constant of 3.07 emuK/gOe. The effective magnetic moment decreased with temperature suggesting an antiferromagnetic behavior when MIL was frozen. (**D**) Ordering transition in magnetic susceptibility from paramagnet to antiferromagnet when MIL loading was at 80%. An upward cusp in the susceptibility marked the antiferromagnetic transition and represented Neel temperature (T_N) of around 4.1 K with the applied magnetic field of 0.5 kOe.

Supplementary Tables

Table S1. A comparison among this work, various reported self-healable magnetogels and freestanding MIL-based ionogels.

Table S2. Detailed fabrication recipe of various urethane group-based polymers and MINEs.

	PTMEG	DMG	Glycerol	IPDI	MIL
Low-crosslinked polymer	4 mmol	4 mmol	2 mmol	11 mmol	/
Medium-crosslinked polymer	4 mmol	4 mmol	5 mmol	15.5 mmol	/
High-crosslinked polymer	4 mmol	4 mmol	7 mmol	18.5 mmol	/
MINE1	80 wt% (Low-crosslinked polymer)				20 wt%
MINE2	60 wt% (Medium-crosslinked polymer)				40 wt%
MINE3	50 wt% (High-crosslinked polymer)				50 wt%
MINE4	40 wt% (High-crosslinked polymer)				60 wt%
MINE5	30 wt% (High-crosslinked polymer)				70 wt%
MINE6	20 wt% (High-crosslinked polymer)				80 wt%

Table S3. The control of various numeric digits on the display.

Switch 1	Switch 2	Switch 3	Switch 4	Displayed Digit
0	0	0	0	**0**
1	0	0	0	**0**
0	1	0	0	**1**
0	0	1	0	**2**
0	0	0	1	**3**
1	0	1	0	**4**
0	1	1	0	**5**
0	0	1	1	**6**
1	0	1	0	**7**
1	0	0	1	**8**

Note: '1' and '0' denote the 'on' and 'off' status of the switches.

Supplementary Notes

Note S1. Gel permeation chromatography (GPC) analysis of various urethane group-based polymers and MINEs.

GPC characterization was used to estimate molecular weights of as-synthesized urethane group-based polymers and MINEs. Tetrahydrofuran (THF) was the eluent for low-crosslinked and medium-crosslinked polymers, while a 10 mM lithium bromide (LiBr)/N,N-dimethylformamide (DMF) solution was used as an eluent for the high-crosslinked polymer. The flow rates for the two eluents were 0.3 and 0.1 mL/min at 50 ℃, respectively. Calibration was performed based on polystyrene standards. Relevant results are shown in table S4.

Table S4. Molecular weights of various urethane group-based polymers and MINEs.

Samples	M_n (Da)	M_w (Da)	PDI
Low-crosslinked polymer	11,522	23,510	2.040504
Medium-crosslinked polymer	15,422	46,511	3.015812
High-crosslinked polymer (soft part)	8,149	34,745	4.263721
High-crosslinked polymer (hard part)	1,511,812	1,586,786	1.049592
MINE with 20 wt% MIL	18,072	35,018	1.937699
MINE with 40 wt% MIL	24,352	46,565	1.912160

Note: Both MINEs with 20 wt% and 40 wt% MIL involved the low-crosslinked polymer. M_n and M_w are number-averaged molecular weight and weight-averaged molecular weight; PDI is polydispersity index, as a measure of homogeneity of molecular weight distribution. $PDI = \frac{Mw}{Mn}$.

The number-averaged (M_n) and weight-averaged molecular weights (M_w) of urethane group-based polymers increase with the higher glycerol and IPDI monomer contents

in the polymers. This shows that polymer chains are longer with more glycerol and IPDI. Glycerol acts as a chain extender and crosslinker, which could control the crosslinking degree and thus, tune the mechanical properties of the polymer. With a higher concentration of glycerol and IPDI, the polydispersity index (*PDI*) of polymers became higher, indicating there are regions with high and low polymeric molecular weights. This is apparent in the high-crosslinked polymer, where there are soft and hard regions in a batch of the polymer.

However, the abovementioned phenomenon diminishes when MIL is added to the polymers. In MINE1 containing 20 wt% MIL, the addition of MIL increases both the Mn and Mw, while decreasing the PDI. This suggests that MIL may enhance the polymerization of polymers. [Emim][$FeCl_4$] could act as a Lewis acidic ionic liquid which accelerates the polymerization rate (*13*, *63*) in the urethane group-based polymer. Note that the medium-crosslinked and high-crosslinked polymers mixed with MILs were hard to dissolve in solvents, and hence their GPC results cannot be obtained.

Note S2. DFT calculation of electrostatic potential map of the urethane group-based polymer

We carried out DFT calculations to estimate the electrostatic potential distribution of the urethane group-based polymer. fig. S42 depicts the electrostatic potential map of the polymer oligomer, where yellow electron clouds represent regions of high electron density, with their size indicative of their electrostatic attraction capacity. After optimization, the distribution of electrons on O and N atoms led to the formation of negative dipoles in the polymer. These dipoles could facilitate the formation of ion-dipole interactions between the polymer and MIL.

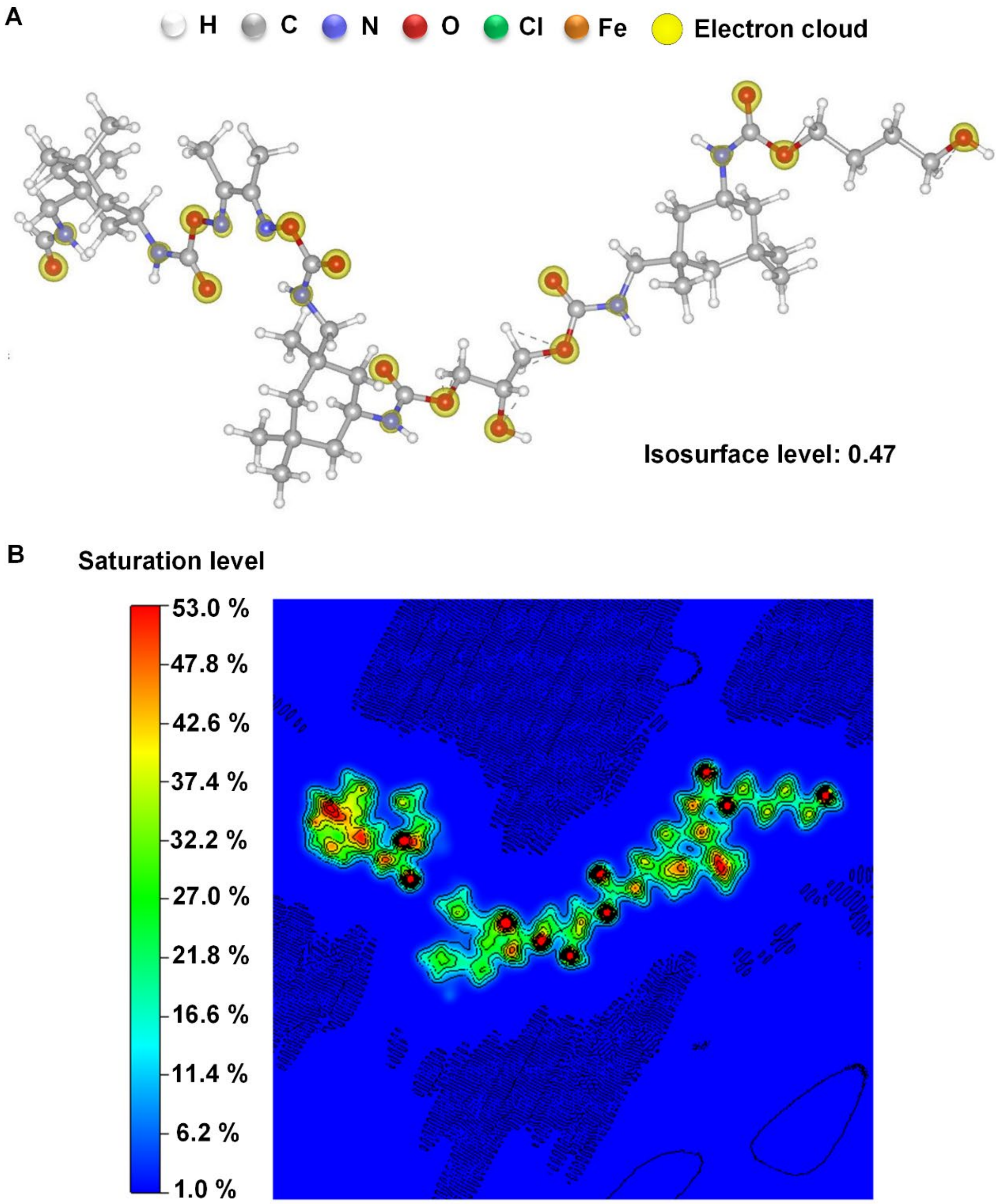

Fig. S42. **Electrostatic potential mapping of the urethane group-based polymer moiety after DFT simulated calculation.** (**A**) Optimized calculation of the polymer moiety through using a DFT. (**B**) 2D data display of (A). Electrons were mainly distributed to O and N atoms, which indicates that there are partial charges on the polymer molecule.

Note S3. Density Functional Theory (DFT) calculations of binding energy (E_b), between the urethane group-based polymer and [Emim][$FeCl_4$], and between the urethane group-based polymer and [Emim][TFSI]

DFT calculations were used to compute the binding energy between urethane group-based polymer and MIL ([Emim][$FeCl_4$]). The results were compared with a commonly used ionic liquid [Emim][TFSI], where they have the same cation but a different anion. Here, the polymer oligomer (y = 1) with PTMEG oligomer (x = 1) was chosen for DTF calculations to investigate the interactions between PUE and the ion pairs. Based on the equation (1) below, the binding energy (E_b) was calculated for the two cases.

$$E_b = E(PUE\ with\ [Emim][anion]) - E(PUE) - E([Emim][anion] \qquad (1)$$

The more negative the E_b, the stronger the interaction between the polymer oligomer and the ionic liquid. Three possible positions of the ionic liquids are shown in fig. S43. Each optimized orientation shows that there is more charge exchange and larger interaction between the polymer and [Emim][$FeCl_4$] than [Emim][TFSI].

All relevant E_b results for the two cases are shown in table S5. The calculation results show that the binding energy for [Emim][$FeCl_4$] was more negative than that of [Emim][TFSI]. Thus, [Emim][$FeCl_4$] has a much higher affinity towards the polymer than [Emim][TFSI], indicating the strong interaction of MIL with [$FeCl_4$]$^-$.

Table S5. Binding energy calculations between the urethane group-based polymer oligomer and each ionic liquid using DFT.

Simulated orientations (oligomer with [Emim][$FeCl_4$])	E_b (eV)	Simulated orientations (oligomer with [Emim][TFSI])	E_b (eV)
Orientation 1	-7.51E-01	Orientation 1	-1.59E-01
Orientation 2	-1.53E-01	Orientation 2	-0.35E-01
Orientation 3	-2.20E-01	Orientation 3	-1.33E-01

Note: E_b is the binding energy between a polymer oligomer and an ionic liquid. The more negative the E_b values, the stronger the interaction between the ionic liquid with the polymer chain.

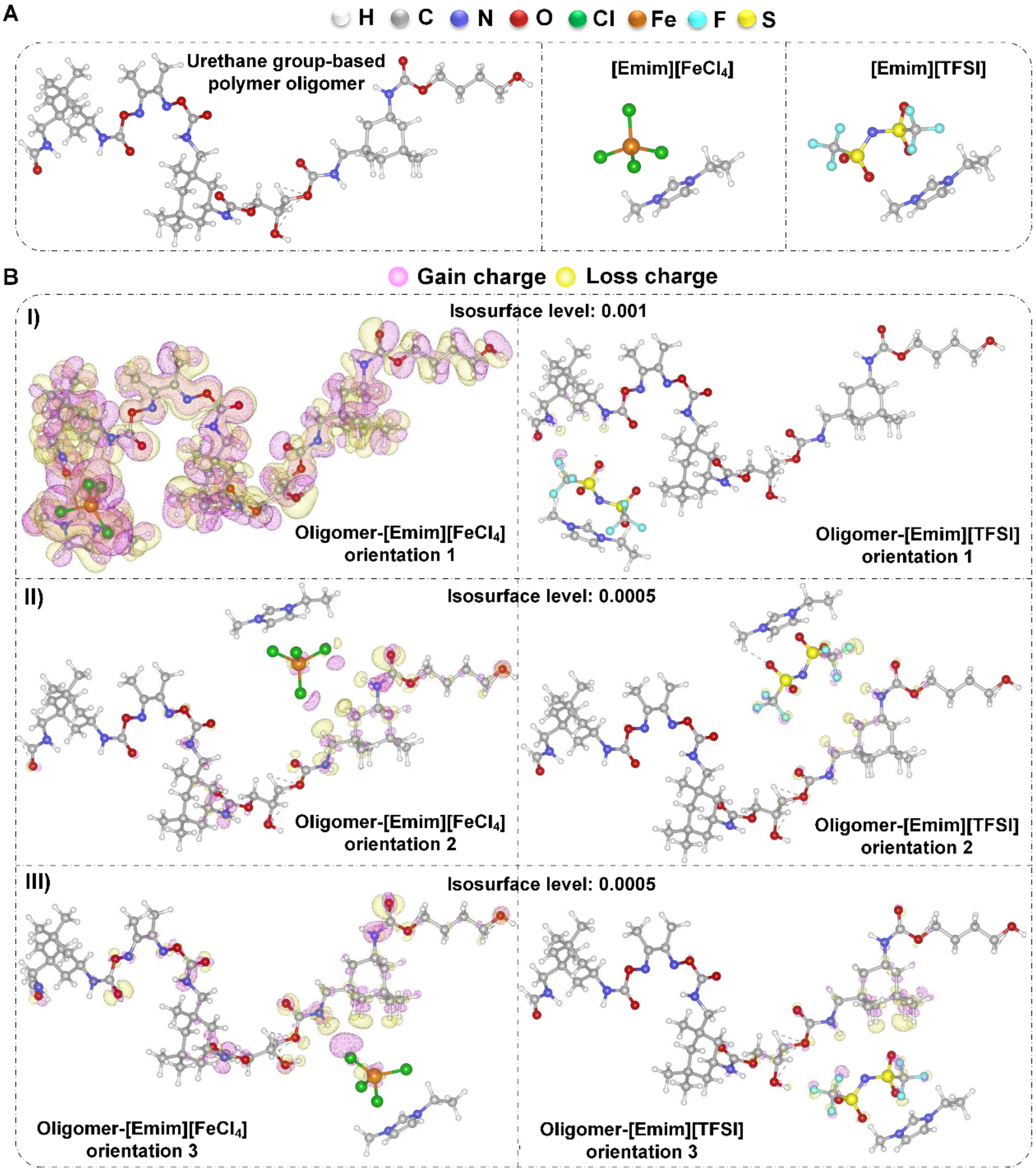

Fig. S43. Three possible orientations of PUE moiety and each ionic liquid for their DFT calculations of binding energy. (**A**) 3D modeling of PUE, [Emim][$FeCl_4$], and [Emim][TFSI]. (**B**) Three optimized orientations: (I) orientation 1, (II) orientation 2, and (III) orientation 3 showing interactions between PUE and each ionic liquid.

Note S4. DFT calculations of interatomic distances of potential hydrogen bonds between the urethane group-based polymer and [Emim][$FeCl_4$].

Using DFT calculations, we delved deeper into the interaction between the urethane group-based polymer and $FeCl_4$ anion in MIL. We divided the long polymer oligomer chain into three main moieties (IPDI-DMG moiety 1, IPDI-Glycerol moiety 2, and IPDI-PTMEG moiety 3). Two simulated orientations of each moiety with [Emim][$FeCl_4$] were optimized as shown in fig. S44.

The calculations showed that some interatomic distances between (C/N-) H from the polymer and Cl from MIL fell within the range of defined hydrogen bonds (2.7-3.3 Å) (*17*). The amide units from the polymer chains are good hydrogen donors, which allows them to establish an intermolecular N-H…Cl hydrogen bonds with Cl from the anion in MIL acting as hydrogen acceptors. Some studies have also reported the formation of C-H…Cl potential hydrogen bonds in paramagnetic ionic liquids (*14*, *64*, *65*). Thus, we showed that potential hydrogen bonds exist between the polymer and the $FeCl_4$ anion in MIL. Such dynamic interactions are crucial in giving MINE its multifunctionality.

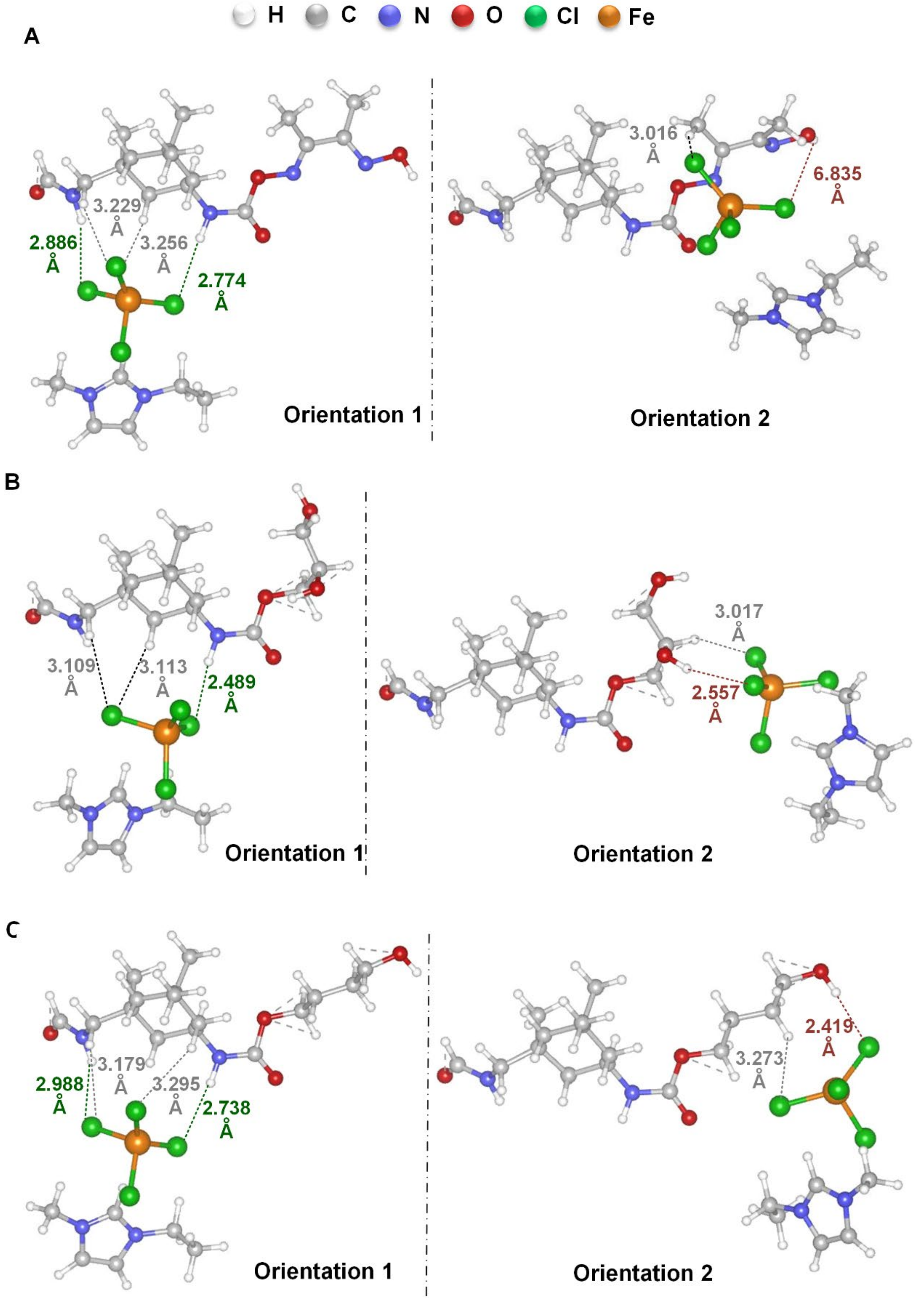

Fig. S44. **DFT calculations of potential hydrogen bonds of three different polymer moieties (IPDI-DMG, IPDI-Glycerol, and IPDI-PTMEG) and [Emim][$FeCl_4$].** (**A**) Interatomic distances of potential hydrogen bonds between IPDI-DMG moiety 1

and MIL under two simulated orientations. (**B**) Interatomic distances of potential hydrogen bonds between IPDI-Glycerol moiety 2 and MIL under two possible orientations. (**C**) Interatomic distances of potential hydrogen bonds between IPDI-PTMEG moiety 3 and MIL under two simulated molecular orientations.

Note S5. The influence of magnetic ionic liquids on hyperelasticity and resilience of soft and self-healable MINEs.

In this case, the as-prepared urethane group-based polymer exhibits certain mechanical properties and autonomous self-healing at room temperature, attributed to both intermolecular hydrogen bonds and intramolecular dimethylglyoxime-urethane groups. Specifically, for the low-crosslinked polymer, its relatively soft and flexible networks enable recovery during the unloading process. In contrast, the high-crosslinked polymer features harder and more brittle networks, potentially leading to the fracture of polymer chains during stretching and thereby preventing recovery (Fig. 3).

The normalized elastic recovery (ε_R) is quantitatively equal to the $(\varepsilon_{max} - \varepsilon_{min})/\varepsilon_{max} \times 100$ during the unloading process of the first cycle, where ε_{max} represents the initial maximum strain of the sample (100% of strain), and ε_{min} corresponds to the residual stain (plastic deformation) (*66*). For the single stretching-releasing cycle, the area beneath the loading curve represents the input energy (U) during stretching, whereas the area beneath the unloading curve represents the elastic recovery energy (E_R). Thus, the dissipation energy (E_D) during the process is the area of the hysteresis loop between the loading and unloading curves. The resilience can be quantitatively described as the ratio of (E_R/U) during the first cycle (*67*, *68*).

Compared to low-crosslinked polymer, the high-crosslinked polymer shows a larger dissipation energy (E_{D2}) and smaller elastic energy (ε_{R2}) during the first loading-unloading cycle which indicates inferior mechanical elasticity. However, the addition of MIL significantly improves their hyperelasticity due to the presence of multiple reversible bonds between the polymer and MIL, including potential hydrogen bonds, metal-coordination bonds, and ion-dipole interactions. When the crosslinking degree of polymers was increased, more urethane groups were formed, facilitating more MIL loading, which led to more reversible bonds. These intermolecular dynamic bonds

break and reform readily when being stretched and released. In addition, MIL acts as a plasticizer to improve the mobility of polymer chains, allowing the molecules to rearrange themselves after being deformed. Hence, the MINE with higher MIL content has smaller dissipation energy (E_{D4}) under the same strain and contributes to elastic recovery (larger ε_{R4}) after being deformed, indicating a more resilient behavior. Fig. S25D illustrates the correlation between the resilience and elastic recovery of MINE and the MIL concentration in its system. Notably, during the first cycle, the resilience and elastic recovery of MINEs paradoxically increase with MIL contents, despite the plasticizing effect of MIL.

Note S6. DFT calculations of various urethan group-based polymer moieties with [Emim][$FeCl_4$] under an electric field.

We further used DFT calculations to verify the confinement of [$FeCl_4$] anion within the MINE framework. Based on the calculation results of three main moieties (IPDI-DMG moiety 1, IPDI-Glycerol moiety 2, and IPDI-PTMEG moiety 3) interacting with [Emim][$FeCl_4$] (shown in Fig. Note 4.1), we applied an electric field (E-field) to re-optimize their structures. In this case, the magnitude of the E-field was set to 0.1 V/Å, and its direction was along the direction of the third lattice vector (IDIPOL=3, DIPOL=0.5 0.5 0.5). The simulated orientations 1 of each moiety with [Emim][$FeCl_4$] after re-optimization are shown in Fig. 2E and fig. S45.

The electric field affected the Emim cation much more than the $FeCl_4$ anion, according to the calculations. This phenomenon is particularly evident for the IPDI-DMG moiety 1 and IPDI-Glycerol moiety 2. Under the E-field, the change in interatomic distances between the N atom from the Emim cation and the N atom from the IPDI moiety was significantly larger than the change observed for the $FeCl_4$ anion and the IPDI moiety. This suggests that the combined effect of potential hydrogen bonds (PHBs) and metal-coordination bonds (MCBs) effectively confines the $FeCl_4$ anions, limiting their movement under the applied electric field. In contrast, the Emim cations exhibit greater mobility within the MINE system due to weaker ion-dipole interactions.

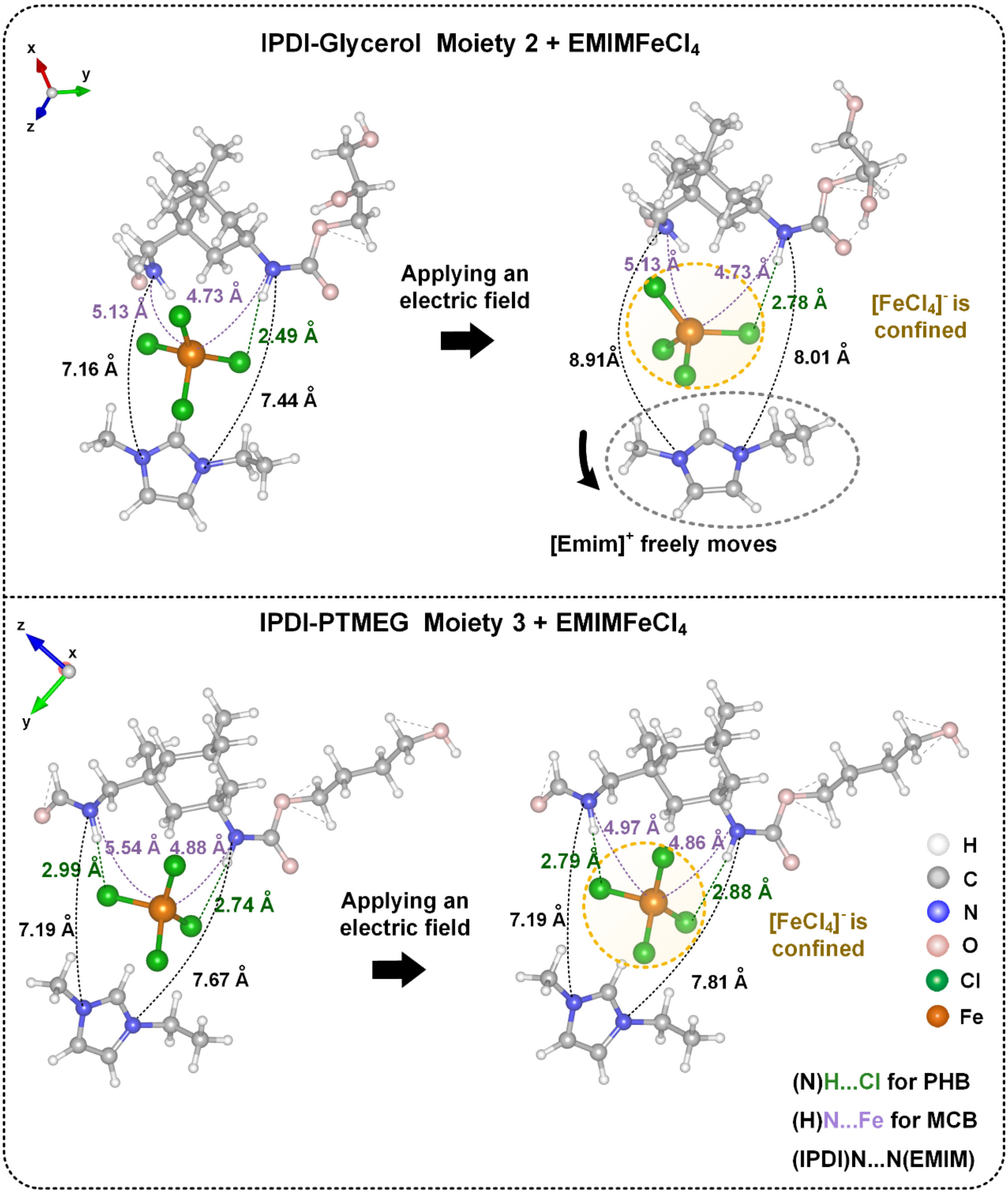

Fig. S45. Under an electric field of 0.1 V/Å, re-optimized structures of different urethane group-based polymer moieties (IPDI-Glycerol and IPDI-PTMEG) and $[Emim][FeCl_4]$ are calculated by DFT simulations.

Movie S1.

The influence of MIL content on magneto-responsive actuation of MINE.

Movie S2.

Comparison of MINE and Fe_3O_4-based composite for multi-functionalities.

Movie S3.

Comparison of elasticity of pure urethane group-based polymer and MINE.

Movie S4.

The influence of MIL content on elasticity performance of MINEs.

Movie S5.

Demonstration of a non-contact magneto-iono-switch system based on MINE for a seven-segment display.

Movie S6.

Demonstration of a MINE-integrated 3D control panel for *8 Balls Pool* game control.

Movie S7.

Demonstration of a hyperelastic magneto-iono-muscle to control a magneto-iono-carrier system for cargo delivery.

REFERENCES AND NOTES

1. E. J. Warrant, Unravelling the enigma of bird magnetoreception. *Nature* **594**, 497–498 (2021).

2. W. Wang, J. V. I. Timonen, A. Carlson, D.-M. Drotlef, C. T. Zhang, S. Kolle, A. Grinthal, T.-S. Wong, B. Hatton, S. H. Kang, S. Kennedy, J. Chi, R. T. Blough, M. Sitti, L. Mahadevan, J. Aizenberg, Multifunctional ferrofluid-infused surfaces with reconfigurable multiscale topography. *Nature* **559**, 77–82 (2018).

3. Y. Kim, H. Yuk, R. Zhao, S. A. Chester, X. Zhao, Printing ferromagnetic domains for untethered fast-transforming soft materials. *Nature* **558**, 274–279 (2018).

4. Y. Zhou, X. Zhao, J. Xu, Y. Fang, G. Chen, Y. Song, S. Li, J. Chen, Giant magnetoelastic effect in soft systems for bioelectronics. *Nat. Mater.* **20**, 1670–1676 (2021).

5. Z. Chen, Y. Wang, H. Chen, J. Law, H. Pu, S. Xie, F. Duan, Y. Sun, N. Liu, J. Yu, A magnetic multi-layer soft for on-demand targeted adhesion. *Nat. Commun.* **15**, 644 (2024).

6. Y. Cao, Y. J. Tan, S. Li, W. W. Lee, H. Guo, Y. Cai, C. Wang, B. C.-K. Tee, Self-healing electronic skins for aquatic environments. *Nat. Electron.* **2**, 75–82 (2019).

7. W. Zhang, B. Wu, S. Sun, P. Wu, Skin-like mechanoresponsive self-healing ionic elastomer from supramolecular zwitterionic network. *Nat. Commun.* **12**, 4082 (2021).

8. Y. J. Tan, H. Godaba, G. Chen, S. T. Tan, G. Wan, G. Li, P. M. Lee, Y. Cai, S. Li, R. F. Shepherd, J. S. Ho, B. C. Tee, A transparent, self-healing and high-κ dielectric for low-field-emission stretchable optoelectronics. *Nat. Mater.* **19**, 182–188 (2020).

9. Y. Kim, X. Zhao, Magnetic soft materials and robots. *Chem. Rev.* **122**, 5317–5364 (2022).

10. S. Hayashi, H.-O. Hamaguchi, Discovery of a magnetic ionic liquid [bmim][$FeCl_4$]. *Chem. Lett.* **33**, 1590–1591 (2004).

11. Z.-L. Xie, A. Jeličić, F.-P. Wang, P. Rabu, A. Friedrich, S. Beuermann, A. Taubert, Transparent, flexible, and paramagnetic ionogels based on PMMA and the iron-based ionic

liquid 1-butyl-3-methylimidazolium tetrachloroferrate(III) [Bmim][$FeCl_4$]. *J. Mater. Chem.* **20**, 9543–9549 (2010).

12. K. Zehbe, M. Kollosche, S. Lardong, A. Kelling, U. Schilde, A. Taubert, Ionogels based on poly(methyl methacrylate) and metal-containing ionic liquids: Correlation between structure and mechanical and electrical properties. *Int. J. Mol. Sci.* **17**, 391 (2016).

13. J. Gao, J.-Q. Wang, Q.-W. Song, L.-N. He, Iron(III)-based ionic liquid-catalyzed regioselective benzylation of arenes and heteroarenes. *Green Chem.* **13**, 1182–1186 (2011).

14. A. García-Saiz, I. Pedro, O. Vallcorba, P. Migowski, I. Hernández, L. F. Barquin, I. Abrahams, M. Motevalli, J. Dupont, J. A. Gonzalez, J. R. Fernández, 1-Ethyl-2,3-dimethylimidazolium paramagnetic ionic liquids with 3D magnetic ordering in its solid state: Synthesis, structure and magneto-structural correlations. *RSC Adv.* **5**, 60835–60848 (2015).

15. L. Li, B. Dou, J. Lan, J. Shang, Y. Wang, J. Yu, E. Ren, S. Lin, Scalable sulfonate-coated cotton fibers as facile recyclable adsorbents for the highly efficient removal of cationic dyes. *Cellulose* **29**, 7445–7463 (2022).

16. L. Jiao, J. Li, L. L. Richard, Q. Sun, T. Stracensky, E. Liu, M. T. Sougrati, Z. Zhao, F. Yang, S. Zhong, H. Xu, S. Mukerjee, Y. Huang, D. A. Cullen, J. H. Park, M. Ferrandon, D. J. Myers, F. Jaouen, Q. Jia, Chemical vapour deposition of Fe–N–C oxygen reduction catalysts with full utilization of dense $Fe–N_4$ sites. *Nat. Mater.* **20**, 1385–1391 (2021).

17. D. E. McRee, Ed., *Practical Protein Crystallography* (Academic Press, 1999).

18. B. Yiming, Y. Han, Z. Han, X. Zhang, Y. Li, W. Lian, M. Zhang, J. Yin, T. Sun, Z. Wu, T. Li, J. Fu, Z. Jia, S. Qu, A mechanically robust and versatile liquid-free ionic conductive elastomer. *Adv. Mater.* **33**, 2006111 (2021).

19. C. Jiang, L. Zhang, Q. Yang, S. Huang, H. Shi, Q. Long, B. Qian, Z. Liu, Q. Guan, M. Liu, R. Yang, Q. Zhao, Z. You, X. Ye, Self-healing polyurethane-elastomer with mechanical tunability for multiple biomedical applications in vivo. *Nat. Commun.* **12**, 4395 (2021).

20. Z. Lei, P. Wu, A highly transparent and ultra-stretchable conductor with stable conductivity during large deformation. *Nat. Commun.* **10**, 3429 (2019).

21. T. Li, Y. Wang, S. Li, X. Liu, J. Sun, Mechanically robust, elastic, and healable ionogels for highly sensitive ultra-durable ionic skins. *Adv. Mater.* **32**, 2002706 (2020).

22. Y. Zhang, B. Yang, X. Zhang, L. Xu, L. Tao, S. Lia, Y. Wei, A magnetic self-healing hydrogel. *Chem. Comm.* **48**, 9305–9307 (2012).

23. Y. M. Kim, J. H. Kwon, S. Kim, U. H. Choi, H. C. Moon, Ion-cluster-mediated ultrafast self-healable ionoconductors for reconfigurable electronics. *Nat. Commun.* **13**, 3769 (2022).

24. L. Yang, L. Sun, H. Huang, W. Zhu, Y. Wang, Z. Wu, R. E. Neisiany, S. Gu, Z. You, Mechanically robust and room temperature self-healing ionogel based on ionic liquid inhibited reversible reaction of disulfide bonds. *Adv. Sci.* **10**, 2207527 (2023).

25. W. Zhu, P. Wu, L. Yang, Y. Chang, Y. Chao, H. Li, Y. Jiang, W. Jiang, S. Xun, Pyridinium-based temperature-responsive magnetic ionic liquid for oxidative desulfurization of fuels. *Chem. Eng. J.* **229**, 250–256 (2013).

26. K. D. Clark, O. Nacham, J. A. Purslow, S. A. Pierson, J. L. Anderson, Magnetic ionic liquids in analytical chemistry: A review. *Anal. Chim. Acta* **934**, 9–21 (2016).

27. S. Mugiraneza, A. M. Hallas, Tutorial: A beginner's guide to interpreting magnetic susceptibility data with the Curie-Weiss law. *Commun. Phys.* **5**, 95 (2022).

28. I. de Pedro, D. P. Rojas, J. A. Blanco, J. R. Fernandez, Antiferromagnetic ordering in magnetic ionic liquid Emim [$FeCl_4$]. *J. Magn. Magn. Mater.* **323**, 1254–1257 (2011).

29. L. Liang, Q. Chen, J. Lu, W. Talsma, J. Shan, G. R. Blake, T. T. M. Palstra, J. Ye, Inducing ferromagnetism and Kondo effect in platinum by paramagnetic ionic gating. *Sci. Adv.* **4**, eaar2030 (2018).

30. J. Fan, A. Chen, Studying a flexible polyurethane elastomer with improved impact-resistant performance polymers. *Polymers* **11**, 467 (2019).

31. C. Wang, C. Ma, C. Mu, W. Lin, Tailor-made zwitterionic polyurethane coatings: Microstructure, mechanical property and their antimicrobial performance. *RSC Adv.* **44**, 27522–27529 (2017).

32. X. Yu, Z. Xia, T. Zhao, X. Yuan, L. Ren, Pyrene-enhanced ferromagnetic interaction in a $FeCl_4^-$-based poly(ionic liquid)s organic magnet. *Macromolecules* **54**, 4227–4235 (2021).

33. D. M. Correia, L. C. Fernandes, C. García-Astrain, M. Tariq, J. M. S. S. Esperança, V. de Zea Bermudez, S. Lanceros-Méndez, Magnetic ionic liquid/polymer composites: Tailoring physico-chemical properties by ionic liquid content and solvent evaporation temperature. *Compos. B Eng.* **178**, 107516 (2019).

34. V. I. Petrenko, L. C. Fernandes, O. I. Ivankov, C. R. Tubio, M. Tariq, J. M. S. S. Esperança, D. M. Correia, S. Lanceros-Mendez, Structural organization of ionic liquids embedded in fluorinated polymers. *J. Mol. Liq.* **360**, 119385 (2022).

35. P. Chen, F. Xie, F. Tang, T. McNally, Unexpected plasticization effects on the structure and properties of polyelectrolyte complexed chitosan/alginate materials. *ACS Appl. Polym. Mater.* **2**, 2957–2966 (2020).

36. R. Alves, A. Fidalgo-Marijuan, M. Salado, R. Gonçalves, M. M. Silva, B. Bazán, F. J. del Campo, C. M. Costa, S. Lanceros-Mendez, Agar-based solid polymer electrolyte-containing ionic liquid for sustainable electrochromic devices. *ACS Sustainable Chem. Eng.* **11**, 16575–16584 (2023).

37. J. C. Dias, D. M. Correia, C. M. Costa, G. Botelho, J. L. Vilas-Vilela, S. Lanceros-Mendez, Thermal degradation behavior of ionic liquid/fluorinated polymer composites: Effect of polymer type and ionic liquid anion and cation. *Polymer* **229**, 123995 (2021).

38. M. U. Khan, J. Kim, M. Y. Chougale, C. M. Furqan, Q. M. Saqib, R. A. Shaukat, N. P. Kobayashi, B. Mohammad, J. Bae, H.-S. Kwok, Ionic liquid multistate resistive switching characteristics in two terminal soft and flexible discrete channels for neuromorphic computing. *Microsyst. Nanoeng.* **8**, 56 (2022).

39. J. M. Yun, K. N. Kim, J. Y. Kim, D. O. Shin, W. J. Lee, S. H. Lee, M. Lieberman, S. O. Kim, DNA origami nanopatterning on chemically modified graphene. *Angew. Chem. Int. Ed. Engl.* **51**, 912–915 (2012).

40. J. Barrio, A. Grafmüller, J. Tzadikov, M. Shalom, Halogen-hydrogen bonds: A general synthetic approach for highly photoactive carbon nitride with tunable properties. *Appl. Catal. B* **237**, 681–688 (2018).

41. C.-H. Li, C. Wang, C. Keplinger, J.-L. Zuo, L. Jin, Y. Sun, P. Zheng, Y. Cao, F. Lissel, C. Linder, X.-Z. You, Z. Bao, A highly stretchable autonomous self-healing elastomer. *Nature Chem.* **8**, 618–624 (2016).

42. T. Marshall-Roth, N. J. Libretto, A. T. Wrobel, K. J. Anderton, M. L. Pegis, N. D. Ricke, T. V. Voorhis, J. T. Miller, Y. Surendranath, A pyridinic Fe-N_4 macrocycle models the active sites in Fe/N-doped carbon electrocatalysts. *Nat. Commun.* **11**, 5283 (2020).

43. A. Shigemoto, S. Osaki, M. Mori, K. Tokieda, Magnetic ionic liquid 1-ethyl-3-methylimidazolium tetrachloroferrate characterized by XPS. *Surf. Sci. Spectra* **30**, 014009 (2023).

44. M. Zhang, X. Tao, R. Yu, Y. He, X. Li, X. Chen, W. Huang, Self-healing, mechanically robust, 3D printable ionogel for highly sensitive and long-term reliable ionotronics. *J. Mater. Chem. A* **10**, 12005–12015 (2022).

45. Y. M. Kim, H. C. Moon, Ionoskins: Nonvolatile, highly transparent, ultrastretchable ionic sensory platforms for wearable electronics. *Adv. Funct. Mater.* **30**, 1907290 (2020).

46. Y. Ren, J. Guo, Z. Liu, Z. Sun, Y. Wu, L. Liu, F. Yan, Ionic liquid–based click-ionogels. *Sci. Adv.* **5**, eaax0648 (2019).

47. J. Xu, H. Wang, X. Du, X. Cheng, Z. Du, H. Wang, Highly stretchable PU ionogels with self-healing capability for a flexible thermoelectric generator. *ACS Appl. Mater. Interfaces* **13**, 20427–20434 (2021).

48. J. Xu, H. Wang, X. Du, X. Cheng, Z. Du, H. Wang, Self-healing, anti-freezing and highly stretchable polyurethane ionogel as ionic skin for wireless strain sensing. *J. Chem. Eng.* **426**, 130724 (2021).

49. L. Xu, Z. Huang, Z. Deng, Z. Du, T. L. Sun, Z.-H. Guo, K. Yue, A transparent, highly stretchable, solvent-resistant, recyclable multifunctional ionogel with underwater self-healing and adhesion for reliable strain sensors. *Adv. Mater.* **33**, 2105306 (2021).

50. Z. Huang, Z. Deng, X. Liu, T. Huang, Y. Hu, Y. Chen, Y. Liu, Z.-H. Guo, K. Yue, Highly stretchable, strain-stiffening, self-healing ionic conductors for wearable sensors. *J. Chem. Eng.* **449**, 137633 (2022).

51. Y. Zhang, M. Li, B. Qin, L. Chen, Y. Liu, X. Zhang, C. Wang, Highly transparent, underwater self-healing, and ionic conductive elastomer based on multivalent ion–dipole interactions. *Chem. Mater.* **32**, 6310–6317 (2020).

52. B. Zhao, S. Li, X. Liao, J. Li, W. Ma, Y. Dong, X. Zhou, Y. Liu, Synthesis and properties of magnetic self-healing polymers: An effective method for improving interface compatibility of doped functional polymers. *ChemNanoMat* **5**, 642–650 (2019).

53. F. Gang, H. Yan, C. Ma, L. Jiang, Y. Gu, Z. Liu, L. Zhao, X. Wang, J. Zhang, X. Sun, Robust magnetic double-network hydrogels with self-healing, MR imaging, cytocompatibility and 3D printability. *Chem. Commun.* **55**, 9801–9804 (2019).

54. K. Liu, X. Pan, L. Chen, L. Huang, Y. Ni, J. Liu, S. Cao, H. Wang, Ultrasoft self-healing nanoparticle-hydrogel composites with conductive and magnetic properties. *ACS Sustainable Chem. Eng.* **6**, 6395–6403 (2018).

55. M. Döbbelin, V. Jovanovski, I. Llarena, L. J. C. Marfil, G. Cabañero, J. Rodriguez, D. Mecerreyes, Synthesis of paramagnetic polymers using ionic liquid chemistry. *Polym. Chem.* **2**, 1275–1278 (2011).

56. Z.-L. Xie, D. S. Su, Ionic liquid based approaches to carbon materials synthesis. *Eur. J. Inorg. Chem.* **2015**, 1137–1147 (2015).

57. C. Yu, C.-F. Wang, S. Chen, Robust self-healing host–guest gels from magnetocaloric radical polymerization. *Adv. Funct. Mater.* **24**, 1235–1242 (2013).

58. X. Jiang, N. Yan, M. Wang, M. Feng, Q. Guan, L. Xu, Magnetic nanostructure and biomolecule synergistically promoted Suaeda-inspired self-healing hydrogel composite for seawater evaporation. *Sci. Total Environ.* **830**, 154545 (2022).

59. M. Sun, C. Tian, L. Mao, X. Meng, X. Shen, B. Hao, X. Wang, H. Xie, L. Zhang, Reconfigurable magnetic slime robot: Deformation, adaptability, and multifunction. *Adv. Funct. Mater.* **32**, 2112508 (2022).

60. A. J. Bandodkar, C. S. López, A. M. V. Mohan, L. Yin, R. Kumar, J. Wang, All-printed magnetically self-healing electrochemical devices. *Sci. Adv.* **2**, e1601465 (2016).

61. W. Zhao, M. Zhou, L. Lv, H. Fu, Self-healing, conductive and magnetic $ZnFe_2O_4$/MCNT/PPy ternary composite hydrogels. *J. Alloys Compd.* **886**, 161083 (2021).

62. Z. Zhang, J. T. Heron, A. Pena-Francesch, Adaptive magnetoactive soft composites for modular and reconfigurable actuators. *Adv. Funct. Mater.* **2215248**, (2023).

63. H. Liu, R. Zhao, X. Song, F. Liu, S. Yu, S. Liu, X. Ge, Lewis acidic ionic liquid [Bmim]$FeCl_4$ as a high efficient catalyst for methanolysis of poly (lactic acid). *Catal. Lett.* **147**, 2298–2305 (2017).

64. T. Bäcker, O. Breunig, M. Valldor, K. Merz, V. Vasylyeva, A.-V. Mudring, In-situ crystal growth and properties of the magnetic ionic liquid [C_2mim][$FeCl_4$]. *Cryst. Growth Des.* **11**, 2564–2571 (2011).

65. A. García-Saiz, P. Migowski, O. Vallcorba, J. Junquera, J. A. Blanco, J. A. González, M. T. Fernández-Díaz, J. Rius, J. Dupont, J. R. Fernández, I. de Pedro, A magnetic ionic liquid based on tetrachloroferrate exhibits three-dimensional magnetic ordering: A combined experimental and theoretical study of the magnetic interaction mechanism. *Chem. Eur. J.* **20**, 72–76 (2014).

66. L. Gao, B.-L. Hu, L. Wang, J. Cao, R. He, F. Zhang, Z. Wang, W. Xue, H. Yang, R.-W. Li, Intrinsically elastic polymer ferroelectric by precise slight cross-linking. *Science* **381**, 540–544 (2023).

67. J. Nan, G. Zhang, T. Zhu, Z. Wang, L. Wang, H. Wang, F. Chu, C. Wang, C. Tang, A highly elastic and fatigue-resistant natural protein-reinforced hydrogel electrolyte for reversible-compressible quasi-solid-state supercapacitors. *Adv. Sci.* **7**, 2000587 (2020).

68. G. L. Gregory, G. S. Sulley, J. Kimpel, M. Łagodzińska, L. Häfele, L. P. Carrodeguas, C. K. Williams, Block poly(carbonate-ester) ionomers as high-performance and recyclable thermoplastic elastomers. *Angew. Chem. Int. Ed. Engl.* **61**, e2022107 (2022).
